# Oxidative stress in vascular surgical diseases: mechanisms, impacts and therapeutic perspectives

**DOI:** 10.3389/fphar.2025.1527684

**Published:** 2025-04-09

**Authors:** Haosen Xu, Jin Yang, Zhanhui Wei, Shijie Bao, Zhuo Liu

**Affiliations:** ^1^ Department of Vascular Surgery, China-Japan Union Hospital, Jilin University, Changchun, China; ^2^ College of Basic Medical Sciences, Jilin University, Changchun, China

**Keywords:** oxidative stress, vascular surgery diseases, reactive oxygen species, antioxidant, therapy

## Abstract

The role of oxidative stress in vascular surgical diseases has increasingly been recognized as significant. This paper systematically reviews the specific mechanisms of oxidative stress in a various vascular surgical condition, including aortic dissection, abdominal aortic aneurysm, thrombosis, diabetic foot, and thromboangiitis obliterans, while also exploring related therapeutic strategies. Oxidative stress arises from an imbalance between free radicals and antioxidants, where excess reactive oxygen species and other free radicals can exacerbate inflammatory response. This paper delves into the pathogenic mechanisms of oxidative stress in the aforementioned diseases and discusses potential methods for utilizing antioxidants to reduce oxidative stress levels. Additionally, this paper highlights the challenges faced by current antioxidant therapies and identifies future research directions. By summarizing current research progress, this paper aims to provide a theoretical basis for more effective treatment strategies of vascular surgical diseases, with the hope of advancing the field.

## 1 Introduction

Vascular surgical disorders, as a significant medical challenge profoundly affecting the structure and function of blood vessels, encompass both arterial and venous disorders. These conditions have become important contributors to mortality and disability worldwide. Underlying these diseases are numerous risk factors such as hypercholesterolemia, diabetes mellitus, obesity, hypertension and ageing, many of which insidiously compromise vascular health through the mechanism of oxidative stress (Hulsmans and Holvoet, 2010).

The biological concept of oxidative stress originated from Helmut Sies in 1985 (SIES, 1985), revealing the subtle changes in the balance between pro-oxidants and antioxidants, which often skews towards pro-oxidants, leading to uncontrolled redox signaling and molecular damage. As research has progressed, this concept has been precisely defined as the imbalance between oxidants and antioxidants, with a tendency toward oxidants resulting in severe consequences (Sies et al., 2017). Moderate oxidative stress is crucial for cellular signaling, gene expression regulation, and maintenance of cellular functions; however, once this balance is disrupted, an excess of reactive oxygen species (ROS) [such as superoxide anions (O_2_·−), hydrogen peroxide (H_2_O_2_), and hydroxyl radicals (·OH) singlet oxygen (^1^O_2_)] can impair the redox equilibrium within cells, triggering a cascade of complex biological effects and pathological processes that pose a serious threat to cellular health (Lushchak, 2014).

In the vascular system, nitric oxide (NO) produced by endothelial cells is crucial for maintaining vascular homeostasis. However, excessive O_2_·^−^ act as antagonists to NO, not only reducing its production but also accelerating its degradation, leading to endothelial dysfunction. This dysfunction can result in impaired vasodilation, thrombosis, exacerbated inflammatory responses, and increased vascular permeability (Incalza et al., 2018). Additionally, ROS promote the proliferation and migration of vascular smooth muscle cells, contributing to vascular wall thickening and lumen narrowing. Under oxidative stress, these cells may also secrete pro-inflammatory cytokines and matrix metalloproteinases, further exacerbating vascular wall damage and stenosis (Gomez and Owens, 2012; Wu et al., 2023). Oxidative stress activates various signaling pathways, such as NF-κB, promoting the expression of inflammatory mediators and adhesion molecules, enhancing interactions between leukocytes and endothelial cells. The infiltration and activation of inflammatory cells increase ROS production, creating a vicious cycle that worsens local vascular injury and dysfunction. Moreover, prolonged oxidative stress can lead to structural and functional damage to cellular DNA, proteins, and lipids, potentially triggering apoptosis or necrosis, thereby laying the groundwork for the progression of vascular diseases (Nikkel and Wetmore, 2023).

**FIGURE 1:**
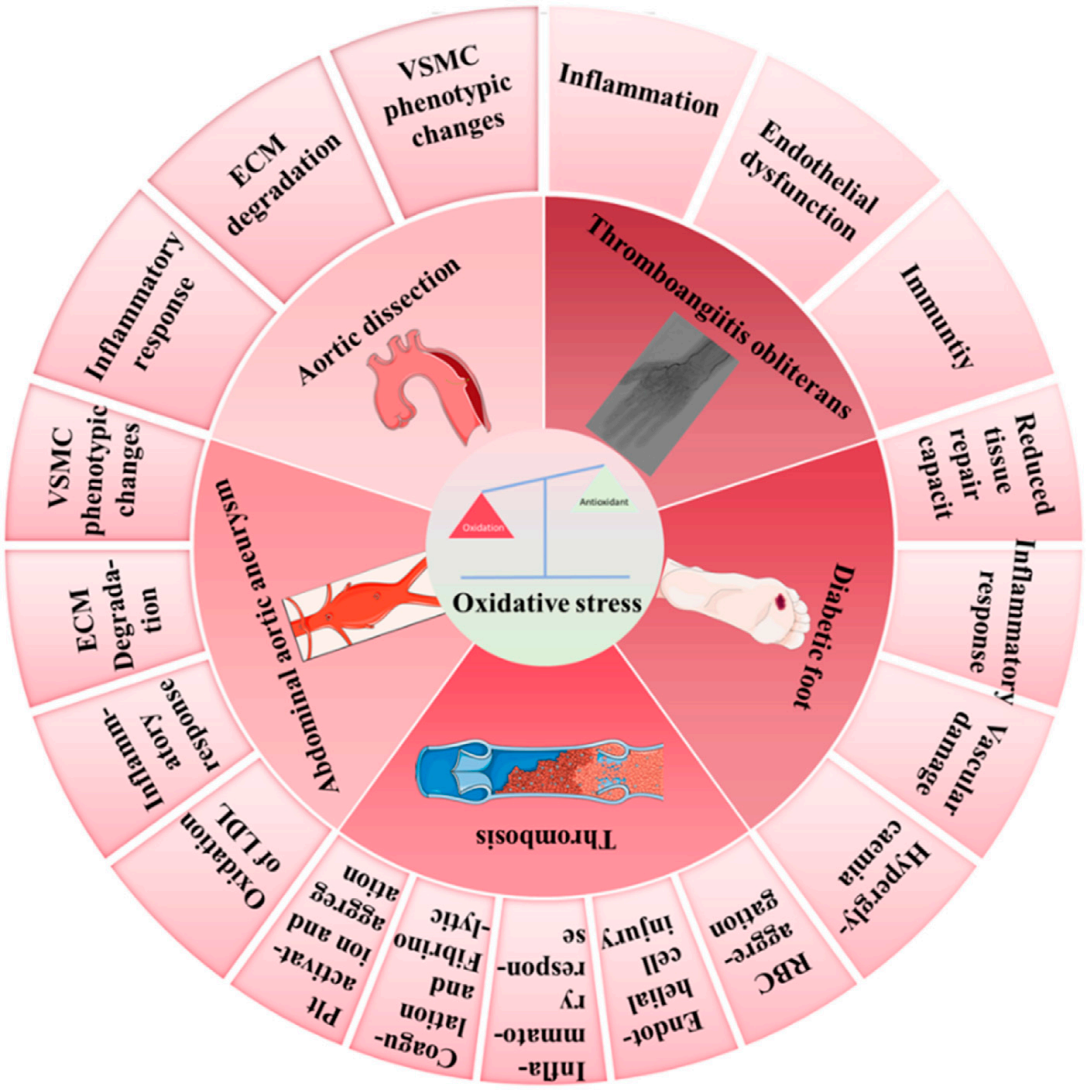
Illustration of various factors leading to imbalance of the oxidative-antioxidative system and the development of cardiovascular and metabolic diseases. (This schematic was created using Servier Medical Art templates, which are licensed under a Creative Commons Attribution 3.0 Unported License; https://smart.servier.com.)

Given the significant role of oxidative stress in vascular surgical diseases, reducing oxidative stress has become one of the key strategies for treating these conditions. This includes enhancing the body’s antioxidant capacity, removing excess oxidants, and repairing tissue damage caused by oxidative stress (Jin and Kang, 2024). Pharmacological treatments featuring antioxidants have shown considerable promise in this field. However, despite research indicating that antioxidants can effectively improve clinical outcomes in cardiovascular diseases, challenges remain in addressing oxidative stress treatment. The complex physiological and pathological mechanisms underlying oxidative stress are not yet fully understood, and the side effects and long-term efficacy of antioxidants require further investigation (Feng et al., 2019). This paper aims to delve into the concept of oxidative stress, the sources and clearance mechanisms of ROS, and to discuss in detail the pathogenic mechanisms and therapeutic strategies related to oxidative stress in vascular surgical diseases such as aortic dissection (AD), abdominal aortic aneurysm (AAA), thrombosis, diabetic foot, and thromboangiitis obliterans. Through this research, we hope to provide more precise and effective treatment strategies for the prevention and management of vascular surgical diseases, ultimately benefiting a larger patient population (Figure 1).

## 2 Vascular surgical diseases and oxidative stress

**FIGURE 2:**
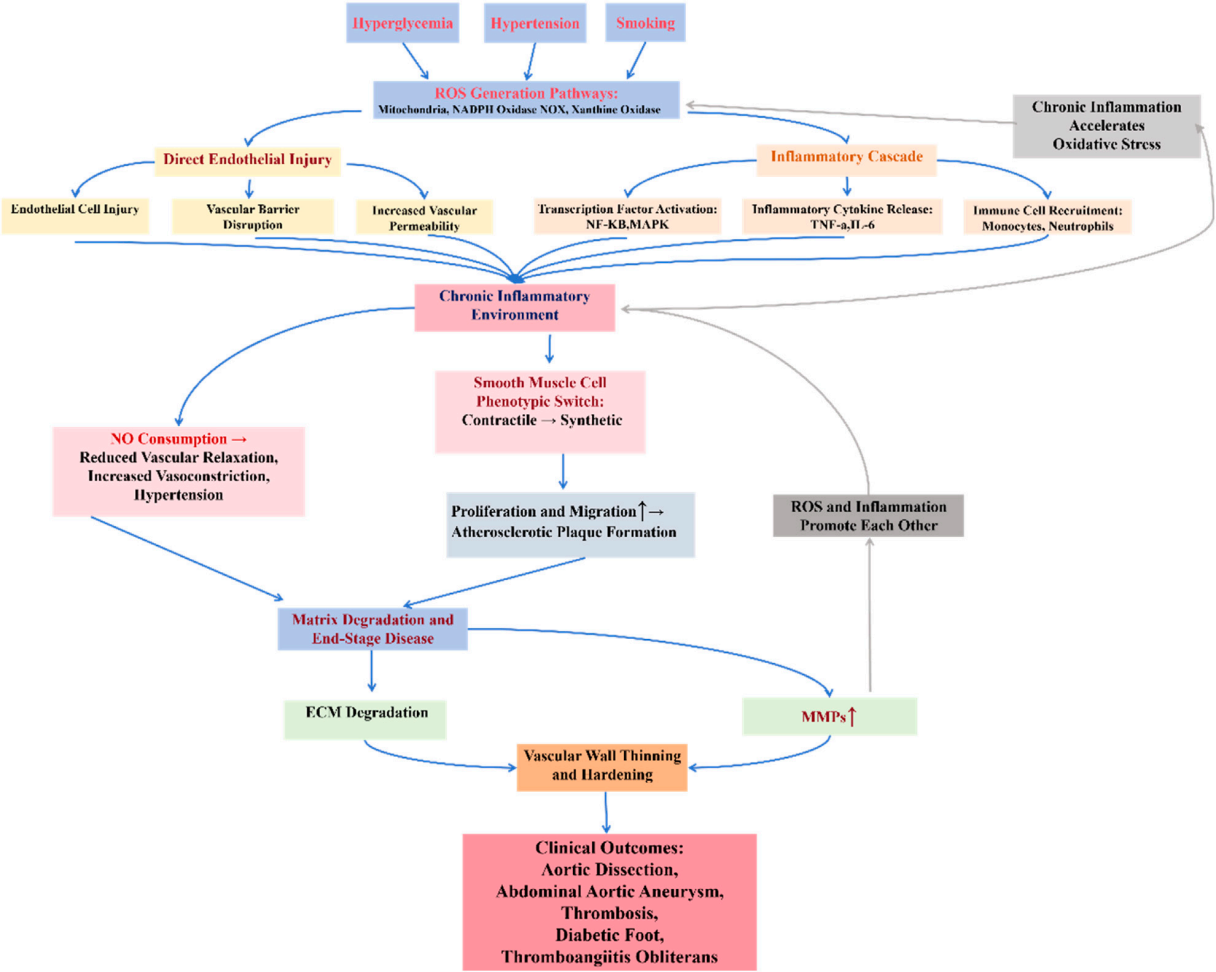
Oxidative stress-inflammation-vascular damage axis.

To establish a unified theoretical framework for understanding the mechanistic role of oxidative stress across vascular pathologies, we propose the “Oxidative Stress-Inflammation-Vascular Injury Axis” (Figure 2). This model can explain how oxidative stress can ultimately lead to vascular damage by activating the inflammatory response, destroying the vascular endothelium, and promoting the phenotypic transformation of smooth muscle cells (VSMCs). When the organism is subjected to risk factors such as hyperglycemia, hypertension, and smoking, the level of ROS is elevated, and ROS mainly originate from mitochondrial, NADPH oxidase (NOX), and xanthine oxidase pathways. Excess ROS can directly damage endothelial cells, disrupt the vascular barrier, and increase vascular permeability, as well as exacerbate the inflammatory response by activating transcription factors such as NF-κB,MAPK and promoting the release of inflammatory factors (e.g., TNF-α, IL-6). Inflammatory factors further attract monocytes and neutrophils to accumulate, creating a chronic inflammatory environment. In addition, ROS deplete nitric oxide (NO), leading to decreased vasodilatory function and promoting vasoconstriction and hypertension. Stimulated by oxidative stress and inflammatory signals, VSMCs shift from a contractile phenotype to a synthetic phenotype, a shift that promotes proliferation and migration of VSMCs, exacerbating atherosclerosis and atheroma formation. Ultimately, inflammation and oxidative stress lead to degradation of the extracellular matrix (ECM) and increased expression of matrix metalloproteinases (MMPs), which accelerates the thinning of the vascular wall and promotes vascular sclerosis, aneurysm rupture, thrombosis, and other diseases.

### 2.1 Role of oxidative stress in AD and related therapeutic strategies

**FIGURE 3:**
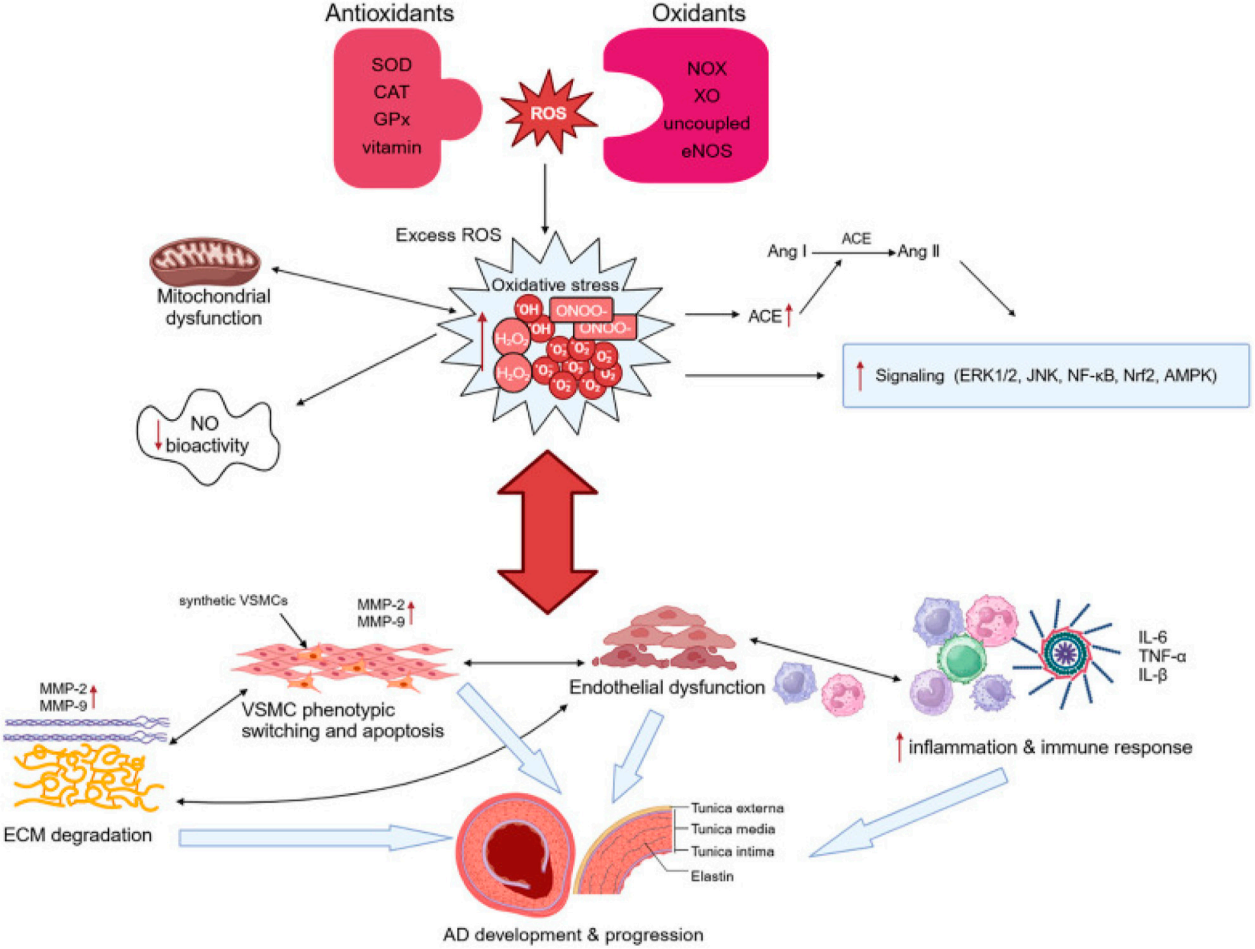
Illustration of pathogenesis induced by oxidative stress in AD. Adapted from reference Xu et al. (2024), Copyright 2024 Frontiers Media S.A. All rights reserved.

AD occurs when a tear forms in the inner wall of the aorta, allowing blood to flow between the layers of the arterial wall and creating a false lumen, which leads to abnormal blood flow and significantly increases the risk of aortic rupture. In this process, oxidative stress plays a crucial role by inducing VSMC phenotypic changes, ECM degradation, and inflammatory responses, thereby promoting the formation and progression of AD (Figure 3).

#### 2.1.1 Oxidative stress and phenotypic changes in VSMC

Under oxidative stress, elevated reactive oxygen species (ROS) drive the transition of vascular smooth muscle cells (VSMCs) from a contractile to a synthetic phenotype, a pivotal event in vascular pathophysiology. Contractile VSMCs maintain vascular tone under physiological conditions, but redox imbalance induced by ROS disrupts this homeostasis, activating key signaling pathways—including MAPK, NF-κB, and TGF-β/Smad—that orchestrate phenotypic reprogramming (Bonetti et al., 2021).

#### 2.1.1.1 MAPK pathway

ROS activate MAPK cascades (ERK1/2, p38 MAPK, JNK) to promote VSMC proliferation and synthetic phenotype acquisition. ERK1/2 activation *via* the RAS/RAF/MEK axis enhances proliferative gene expression (Yorn et al., 2024), while p38 MAPK phosphorylates transcription factors (e.g., ATF2, CREB) to upregulate inflammatory cytokines (IL-6, TNF-α) and matrix metalloproteinases (MMP-9), driving extracellular matrix (ECM) degradation (Zhang et al., 2024). JNK activation through MKK4/MK7 promotes AP-1-mediated transcription of MMPs (MMP-2/9), pro-inflammatory factors (IL-1β), and proliferative markers (PCNA), further accelerating VSMC migration and synthetic transformation (Kigawa et al., 2014; Li and Kong, 2020).

#### 2.1.1.2 NF-κB pathway

ROS-induced activation of the IKK complex leads to IκBα degradation, releasing NF-κB dimers (p65/p50; p52/RelB) that translocate to the nucleus. This triggers the expression of genes involved in inflammation, ECM remodeling, and VSMC proliferation, such as MMP-9, IL-6, TNF-α, and PCNA. MMP-9 promotes ECM degradation, while IL-6 and TNF-α drive inflammation, further stimulating VSMC proliferation and migration. This process contributes to vascular remodeling, a key factor in atherosclerosis and vascular diseases (Ahmed et al., 2017; Csiszar et al., 2008; Jiang and Qian, 2023). The ROS-IKK-NF-κB signaling pathway plays a crucial role in vascular pathology, offering potential targets for therapeutic intervention in cardiovascular diseases.

#### 2.1.1.3 TGF-β/Smad pathway

ROS enhance TGF-β signaling by increasing receptor activation, leading to Smad2/3 phosphorylation. This enables the formation of Smad2/3–Smad4 complexes, which translocate to the nucleus and drive the expression of fibrotic genes like collagen and α-SMA, promoting VSMC synthetic behavior. ROS also activate non-Smad pathways, including ERK and PI3K/Akt, which synergize with TGF-β to further enhance VSMC migration, proliferation, and ECM synthesis (Chen and Chang, 2022; Wang J. et al., 2024). Targeting both Smad-dependent and non-Smad pathways may offer therapeutic opportunities for vascular fibrosis and inflammation.

#### 2.1.1.4 ROS species-specific roles

Superoxide (O_2_^−^): Activates NF-κB/MAPK and reacts with NO to form peroxynitrite (ONOO^−^), amplifying oxidative stress (Takaishi et al., 2021). Hydrogen peroxide (H_2_O_2_): Diffuses across membranes to activate MAPK/NF-κB, promoting phenotypic switching (Velarde et al., 2004). Hydroxyl radical (·OH): Directly damages biomolecules, inducing inflammation and apoptosis (Fujii et al., 2022). Nitric oxide (NO): At low levels, maintains vasodilation; under oxidative stress, reacts with O_2_^−^ to form ONOO^−^, exacerbating VSMC dysfunction (Piacenza et al., 2022).

#### 2.1.1.5 Therapeutic implications

**FIGURE 4:**
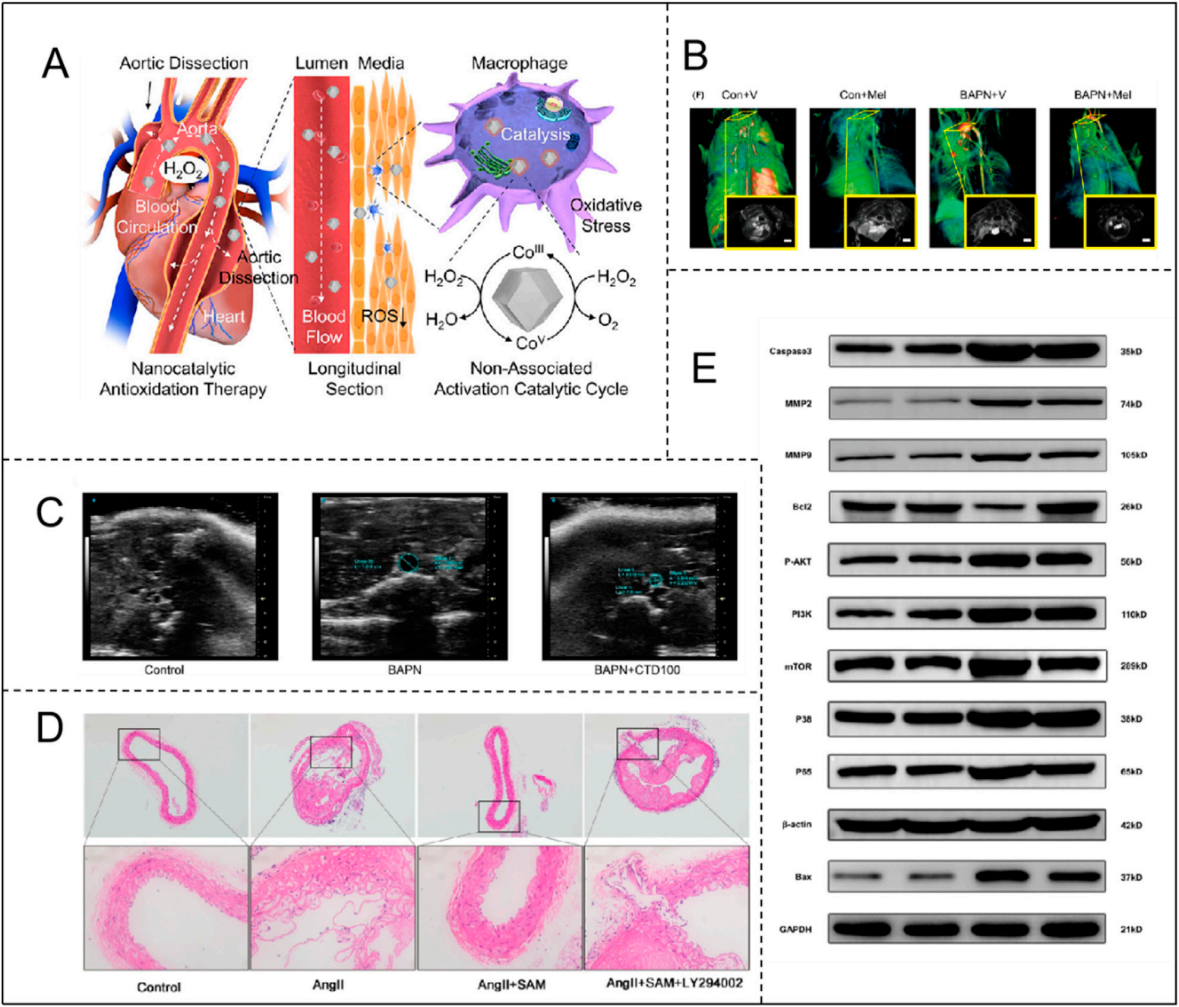
**(A)** Catalytic mechanism of Co-ZIF-8 nanocatalyst and its application in AD. Adapted with permission from Ref. Yang et al. (2024). Copyright (2024) American Chemical Society; **(B)** Therapeutic efficacy of melatonin in BAPN-induced TAAD mice with CT angiographic images of each group. Adapted with permission from Ref. Xia et al. (2020). Copyright (2020) John Wiley & Sons A/S; **(C)** Ultrasonography shows that CTD inhibits the development of BAPN-induced TAD. Adapted with permission from Ref. Han et al. (2024). Copyright (2024) Elsevier B.V.; **(D)** SAM attenuates Ang II-induced AD formation in ApoE^−/−^ mice through a PI3K/AKT-dependent pathway. Adapted with permission from Ref. Shen et al. (2024). Copyright (2023) Elsevier Inc.; **(E)** SEI attenuates BAPN/ANGII-induced apoptosis through the aortic PI3K/Akt/NF-κb pathway. Adapted with permission from Ref. Zhao K. et al. (2023). Copyright (2023) Elsevier B.V.

S-adenosylmethionine (SAM): Suppresses AngII-induced VSMC autophagy and phenotypic switching *via* PI3K/Akt/mTOR activation, mitigating AD progression (Figure 4D; Table 1) (Shen et al., 2024). SAM also enhances glutathione synthesis and reduces homocysteine toxicity (Lim et al., 2011). Coumarin derivatives (e.g., CTD): Inhibit NF-κB to attenuate inflammation, reduce MMP2/9 activity, and preserve ECM integrity in thoracic aortic dissection models (Figure 4C; Table 1) (Han et al., 2024). Sirtuin 3 (Sirt3): Mitigates ROS production, preventing VSMC apoptosis and vascular inflammation (Table 1) (Qiu et al., 2021). This integrated framework underscores oxidative stress as a central driver of VSMC dysregulation, highlighting therapeutic strategies targeting redox signaling to restore vascular homeostasis.

**TABLE 1:** Therapies against oxidative stress in aortic dissection.

Disease	Mechanisms	Treatment	Results	References
Aortic dissection	VSMC phenotypic changes	SAM	Activation of the PI3K/AKT/mTOR signaling pathway to inhibit dysregulated cellular autophagy and phenotypic transition in VSMC	Shen et al. (2024)
		CTD	Inhibition of the NF-κB signaling pathway reduces VSMC apoptosis	Han et al. (2024)
		Sirt 3	Reduces ROS-induced VSMC apoptosis	Qiu et al. (2021)
	ECM degradation	SEI	Inhibition of the PI3K/Akt/NF-κB signaling pathway reduces degradation of collagen and elastin fibers in the ECM	Zhao K. et al. (2023)
		IMD1-53	Reduces NADPH oxidase mRNA and protein expression to reduce VSMC and MMPs activation, inhibits Nox4 expression to reduce ROS generation	Lu et al. (2016)
	Inflammatory response	Melatonin	Inhibits macrophage infiltration and alters the positive feedback loop between the inflammatory response and ECM degeneration	Xia et al. (2020)
		Co-ZIF-8	Promotes M1 macrophage transition to M2 phenotype, upregulates DDIT4 expression, inhibits PI3k-Akt pathway, antioxidant and anti-inflammatory	Yang et al. (2024)

#### 2.1.2 Oxidative stress and ECM degradation

ROS promote the degradation of ECM through multiple mechanisms, primarily involving activation of metallo-matrix proteases (MMPs) and inhibition of tissue-inhibited metalloproteinases (TIMPs). Specifically, ROS can directly oxidize the inactive precursors of MMPs (pro-MMPs), disrupting the thiol-zinc bonds within them, which leads to their conversion from an inactive to an active state (Jeong et al., 2022) In addition, ROS induce the expression of MMPs genes through the upregulation of several signaling pathways, including NF-κB, AP-1, and MAPK. In particular, under oxidative stress, NF-κB is activated and enters the nucleus, binding to the promoter region of MMPs genes and promoting the expression of key MMPs such as MMP-9 (Eble and Rezende, 2014). ROS can also upregulate the activity of NADPH oxidase (NOX) through a positive feedback mechanism, generating more ROS, further activating MMPs and accelerating the degradation process of ECM (Gurung et al., 2020).

Beyond directly activating MMPs, ROS also promote ECM degradation by inhibiting the function of TIMPs. TIMPs are natural inhibitors of MMPs, acting by binding to them to suppress their activity. However, under oxidative stress, ROS reduced the expression levels of TIMPs and directly oxidize their structures, diminishing their inhibitory effects on MMPs (Qipshidze et al., 2012). This weakened inhibition leads to uncontrolled MMP activity, resulting in the excessive degradation of crucial ECM components such as collagen and elastin. Moreover, ROS indirectly exacerbated the activity of MMPs by inducing a local inflammatory response. ROS activated pro-inflammatory pathways and induced the release of inflammatory factors such as TNF-α and IL-6, and these inflammatory factors further stimulated the expression and activity of MMPs, promoting ECM degradation (Cao et al., 2019). Ultimately, the degradation of ECM leads to the disruption of the structural integrity of the vessel wall, promoting the formation of vascular lesions such as aneurysms.

One study indicated that the degradation of collagen and elastin fibers in the ECM was significantly inhibited in Senkyunolide I (SEI)-treated mice, which may have mitigated the progression of thoracic aortic aneurysm and dissection (TAAD) through inhibition of the PI3K/Akt/NF-κB signaling pathway (Figure 4E; Table 1) (Zhao Y. et al., 2023). Another study found that IMD1-53, a factor regulating oxidative stress, acts by reducing oxidative stress, inflammation, VSMC apoptosis, and matrix metalloproteinase activation by decreasing the mRNA and protein expression of NADPH oxidase and inhibiting its activity. IMD1-53 also inhibits the expression of Nox4, which is the major ROS generator in VSMC and involved in AAA pathogenesis (Table 1) (Lu et al., 2016).

#### 2.1.3 Generation of pro-inflammatory cytokine and mechanisms of inflammatory response

ROS induce the generation of pro-inflammatory cytokines through a series of complex signaling pathways, triggering an inflammatory response. Specifically, ROS first activate the NF-κB signaling pathway. Under oxidative stress, ROS activate IκB kinase (IKK), leading to the release of NF-κB and its translocation into the nucleus, which promoties the transcription of key pro-inflammatory factors, such as TNF-α, interleukin-1β (IL-1β) and interleukin-6 (IL-6) (S. Xu et al., 2024). Additionally,ROS also enhance the expression of pro-inflammatory genes by activating the MAPK pathway, especially JNK and p38 MAPK. The activated AP-1 transcription factor enters the nucleus and further promotes the production of IL-1 and TNF-α (Zhao K. et al., 2023). Moreover, ROS activate NLRP3 inflammasome, promoting the activation of inflammasome and the release of IL-1βand IL-18, which exacerbates local inflammatory response. ROS also initiate the inflammatory response by inducing lipid oxidation to generate oxidized lipids and activating Toll-like receptor (TLR) (Chen and Chang, 2022; Wang Y. et al., 2024). These pro-inflammatory factors not only exacerbate the tissue damage caused by oxidative stress, but also further enhance the generation of ROS through a positive feedback mechanism, forming a vicious circle and leading to more severe inflammatory responses and tissue damage.

Research by Xia et al. indicates that in mice, BAPN induction significantly increases the number of CD68(+) macrophages, while melatonin can inhibit macrophage infiltration, altering the positive feedback loop between the inflammatory response and ECM degeneration, effectively preventing the progression of TAAD (Figure 4B; Table 1) (Xia et al., 2020). On the other hand, Yang and Hu et al. constructed Co-ZIF-8 nanocatalysts, which exhibit efficient antioxidant and anti-inflammatory effects through multiple mechanisms, including promoting the transition of M1 macrophages to the M2 phenotype, upregulating DDIT4 expression, and inhibiting the PI3K-Akt pathway. In a BAPN-induced AD animal model, Co-ZIF-8 nanoparticles significantly alleviated the inflammatory response in the aorta and substantially inhibited aortic dilation, thereby effectively delaying the progression of AD (Figure 4A; Table 1) (Yang et al., 2024).

Currently, the treatment of AD and thoracic aortic aneurysms primarily relies on surgical intervention. However, despite numerous studies indicating the potential benefits of antioxidant therapy in AD, several critical issues remain to be addressed. The foremost issue is that most research has f focused on animal models, lacking large-scale human clinical trials to validate their efficacy and safety. Additionally, there is no consensus on the selection and dosage of antioxidants, and different antioxidants may exhibit distinct mechanisms of action and effects, which requires further studies to determine the optimal treatment regimen. Furthermore, ROS demonstrate dual roles in physiologic and pathologic processes, and excessive suppression may lead to adverse effects. Therefore, future research must delve deeper the mechanism of antioxidant therapy more deeply and optimize treatment strategies. This approach not only aims to slow disease progression but may also provide an effective alternative therapy for patients who are unable to tolerate surgery, thereby enriching the therapeutic options.

### 2.2 Role of oxidative stress in AAA and related therapeutic strategies

**FIGURE 5:**
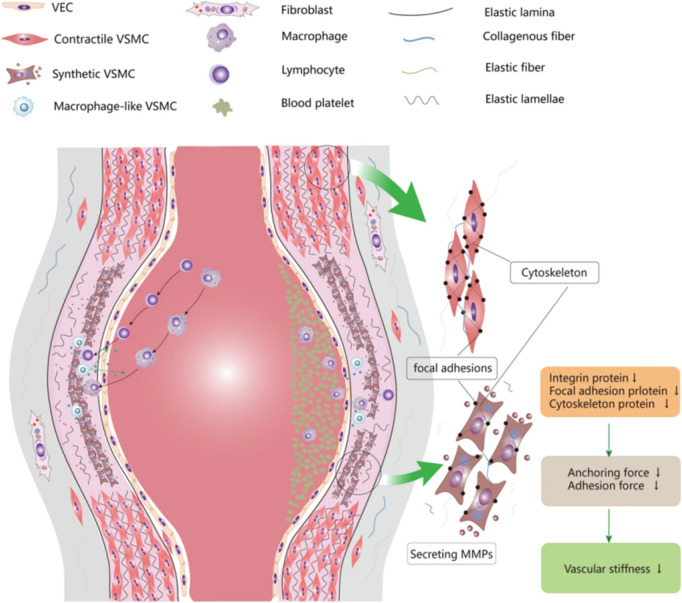
Thoracic aortic aneurysm and oxidative stress. The aortic wall consists of the intima, mid-membrane and ecta, where VSMCs provide contractility. In aortic aneurysms, VSMCs shift to a synthetic phenotype and secrete large amounts of MMP and inflammatory cytokines, leading to downregulation of contractile proteins and ECM catabolism, which weaken active contractility. In addition, macrophage-like VSMCs trigger chronic inflammation and accelerate aortic wall destruction. Adapted with permission from Ref. Cao et al. (2019). Copyright (2022) BioMed Central Ltd.

AAA is characterized by dilatation of the abdominal aorta to more than 1.5 times its normal diameter, involving complex interactions among various cellular and molecular pathways in its pathogenesis. Recent studies increasingly emphasize the central role of oxidative stress in the formation and progression of AAA (Golledge, 2018). In the development of AAA, oxidative stress similarly contributes to the pathologic process by promoting VSMC phenotypic transformation, ECM degradation, and pro-inflammatory cytokine production and inflammatory response, collectively advancing the pathological process. Additionally, AAA formation is facilitated by the accumulation of oxidized low-density lipoprotein (LDL), which recruits and activates inflammatory cells, increases the expression of matrix metalloproteinases (MMPs), and subsequently lead to the degradation of elastin and apoptosis of smooth muscle cells (Figure 5), ultimately resulting in weakening and dilatation of the aortic wall (Kuivaniemi et al., 2015).

**FIGURE 6:**
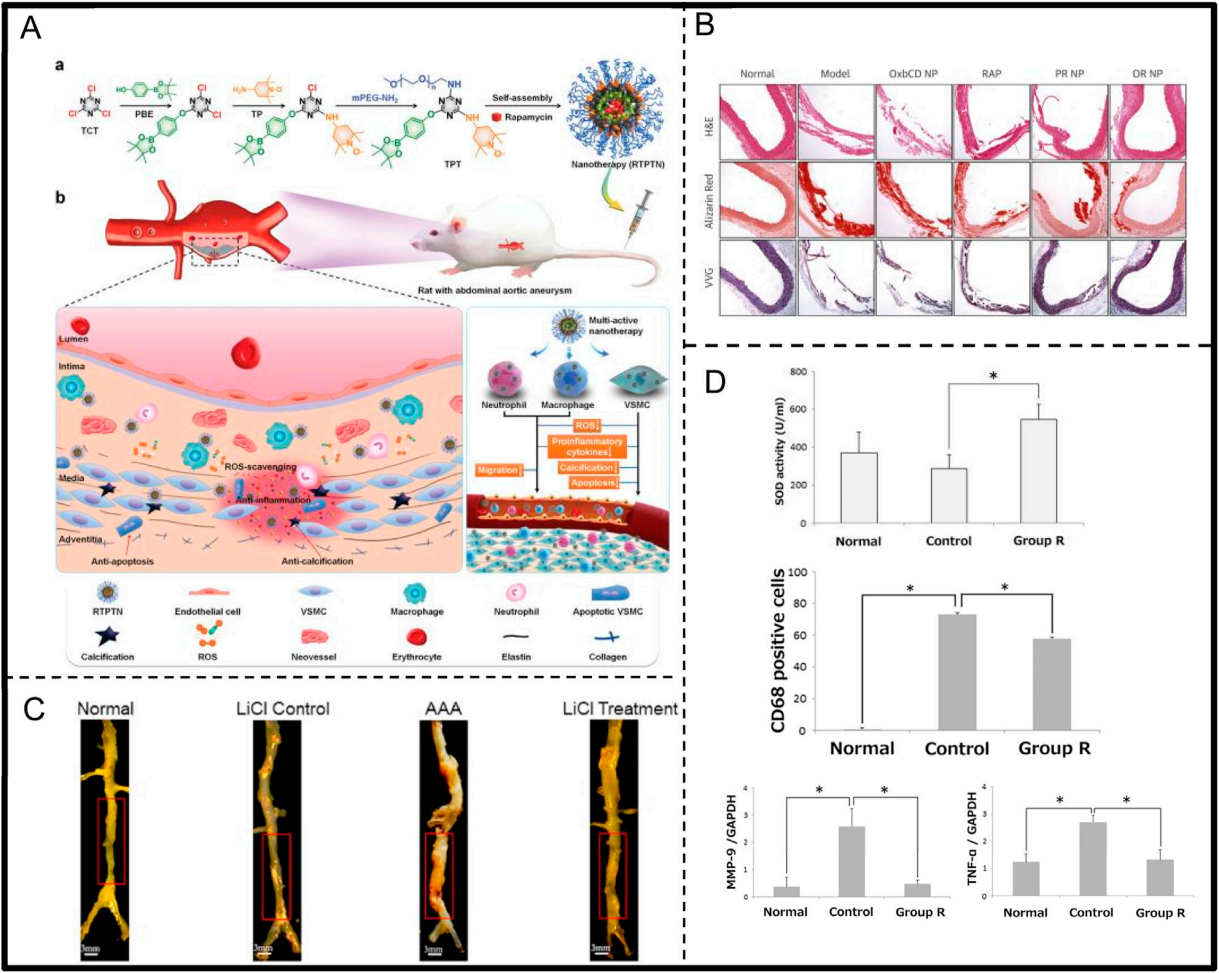
**(A)** TPTN scavenges ROS, alters pathological microenvironment, and targets abdominal aortic aneurysms (AAA)Adapted with permission from Ref. Lin et al. (2022). Copyright (2022) Wiley-VCH GmbH. **(B)** Rapamycin-loaded reactive nanotherapeutics resulted in a significant reduction in the maximum diameter of the abdominal aorta and a decrease in elastin degradation. Adapted with permission from Ref. Cheng et al. (2018). Copyright (2018) Elsevier B.V. **(C)** The diameter of aortic aneurysm in rats in AAA group was significantly enlarged compared with that of normal group, whereas the diameter of the aorta in LiCl-treated group was only slightly enlarged. Adapted with permission from Ref. Xu et al. (2021). Copyright (2021) Elsevier Inc. **(D)** Riboflavin significantly reduces inflammatory responses in AAA. Adapted with permission from Ref. Yu et al. (2016). Copyright (2015) by the Society for Vascular Surgery.

Research by Piechota-Polanczyk et al. demonstrated that simvastatin treatment can reduce the activation of nuclear factor kappa B (NF-κB) and lower the concentration of TNF-α in human AAA wall tissue. Simvastatin also increases catalase (CAT) levels and inhibits lipid peroxidation processes (Table 2) (Piechota-Polanczyk et al., 2012). Yu et al. investigated the effect of oral riboflavin (vitamin B2) in reducing AAA formation in rats by activating endogenous superoxide dismutase (SOD) (Figure 6D; Table 2). The results showed that riboflavin significantly increased SOD activity, reduced ROS and 8-hydroxydeoxyguanosine (8-OHdG)-positive cells, and decreased TNF-α and MMP-9 mRNA expression, ultimately leading to reduced aortic dilatation and increased elastin content, demonstrating the protective role of riboflavin AAA (Yu et al., 2016). Additionally, Alpha-ketoglutarate (AKG) improves AAA by inhibiting the peroxisome proliferator-activated receptor delta/hypochlorous acid/extracellular signal-regulated kinase (PXDN/HOCL/ERK) signalling pathway (Table 2). It was found that AKG improves AAA mitigates oxidative stress and inflammation primarily by suppressing PXDN expression (Liu et al., 2022). Lin et al. synthesized a multifunctional nanocolloid, TPTN, using a “multi-pronged” strategy to treat AAA (Figure 6A; Table 2). This nanomicelle effectively inhibits the migration and activation of inflammatory cells, reduces the generation of inflammatory factors, and protect VSMC from oxidative stress, calcification, and apoptosis, significantly delaying AAA expansion and demonstrating good targeting and efficacy (Lin et al., 2022). Cheng et al. prepared a rapamycin-negative carrier-responsive nanotherapeutic (CROR NP), injected in mice with AAA, CROR NP highly efficiently accumulated in the diseased tissue of AAA, significantly inhibited the migration and activation of inflammatory cells, and regulated a variety of pathological cells, thereby effectively delaying the progression of AAA (Figure 6B; Table 2) (Cheng et al., 2018). In addition. Xu et al. were the first to demonstrate that LiCl could prevent the development of AAA by regulating the glycogen synthase kinase 3 beta/sirtuin 1/nuclear factor kappa B (GSK3β/SIRT1/NF-κB) pathway, inhibiting inflammation, superoxide production and elastin degradation (Figure 6C; Table 2) (Xu et al., 2021).

**TABLE 2:** Therapies against oxidative stress in abdominal aortic aneurysm.

Disease	Mechanisms	Treatment	Results	References
Abdominal Aortic Aneurysm	VSMC phenotypic shift	KMUP-3	Inhibits AngII-induced AAA formation by reducing VSMC phenotypic shift, apoptosis and calcification	Lai et al. (2020)
	Degradation of ECM	Ascorbic acid	Enhances elastin and collagen production in aortic smooth muscle cells while inhibiting inflammatory responses and oxidative stress	Tanaka et al. (2014)
	Inflammatory response	Riboflavin	Activates endogenous SOD, decreases TNF-α and MMP-9 mRNA expression, reduces aortic dilatation and increases elastin content	Yu et al. (2016)
		AKG	Inhibits PXDN/HOCL/ERK signaling pathway, suppresses oxidative stress and inflammatory response	Liu et al. (2022)
		TPTN	Inhibition of inflammatory cell migration and activation, reduction of inflammatory factor production, and VSMC protection	Lin et al. (2022)
		CROR NP	CROR NP efficiently accumulates in diseased tissues of AAA and significantly inhibits migration and activation of inflammatory cells	Cheng et al. (2018)
		LiCl	Inhibits inflammation, superoxide production and elastin degradation by modulating the GSK3β/SIRT1/NF-kB pathway	Xu et al. (2021)
	Oxidation of LDL	Simvastatin	Reduces NF-κB activation and decreases TNF-α concentration in tissues, increases CAT levels and inhibits lipid peroxidation	Piechota-Polanczyk et al. (2012)

### 2.3 Role of oxidative stress in thrombosis and related therapeutic strategies

**FIGURE 7:**
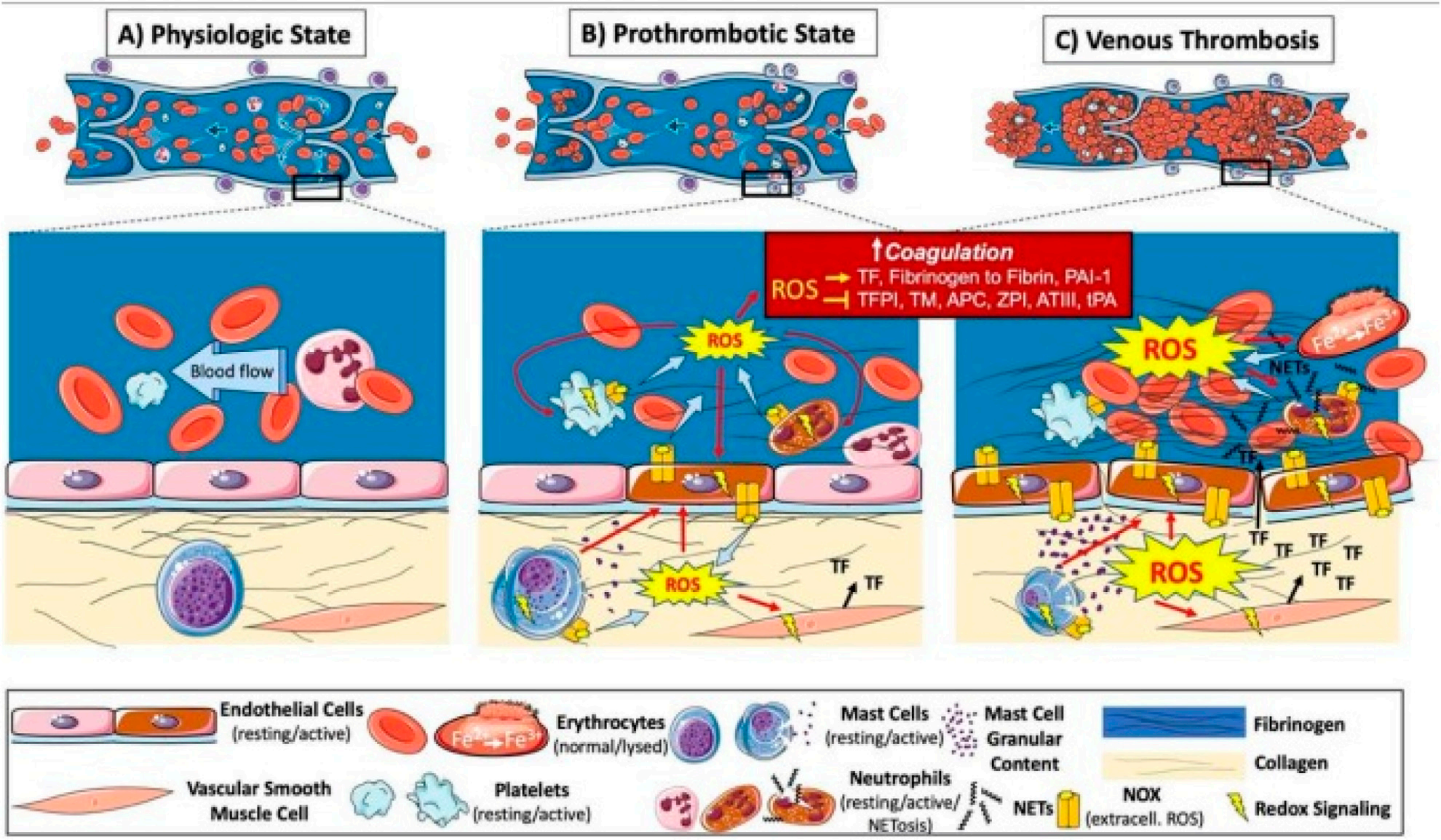
Illustration of pathogenesis induced by oxidative stress in venous thrombosis. ROS are increased at sites of blood stasis and activated endothelial cells promote the recruitment and activation of leukocytes, platelets, mast cells, and vascular smooth muscle cells. These cells activate the coagulation system and platelet aggregation *via* redox signaling, leading to thrombosis. Oxidative stress also promotes hemoglobin oxidation and enhances thrombus stability *via* neutrophil extracellular traps (NETs), further exacerbating oxidative stress and thrombosis. Adapted from reference Gutmann et al. (2020), Int. J. Mol. Sci. 2020 All rights reserved.

Thrombosis refers to the abnormal coagulation of blood within blood vessels, leading to the formation of solid blood clots. This process can occur in both the arterial and venous system and is a core pathologic basis for numerous cardiovascular and peripheral vascular diseases. In this complex pathophysiological evolution, oxidative stress plays a crucial role, profoundly influencing thrombosis formation and development through a series of molecular and cellular mechanisms, including platelet activation and aggregation, activation of coagulation factors, and damage to endothelial cells (Figure 7) (Gutmann et al., 2020).

#### 2.3.1 Mechanisms of platelet activation and aggregation

**FIGURE 8:**
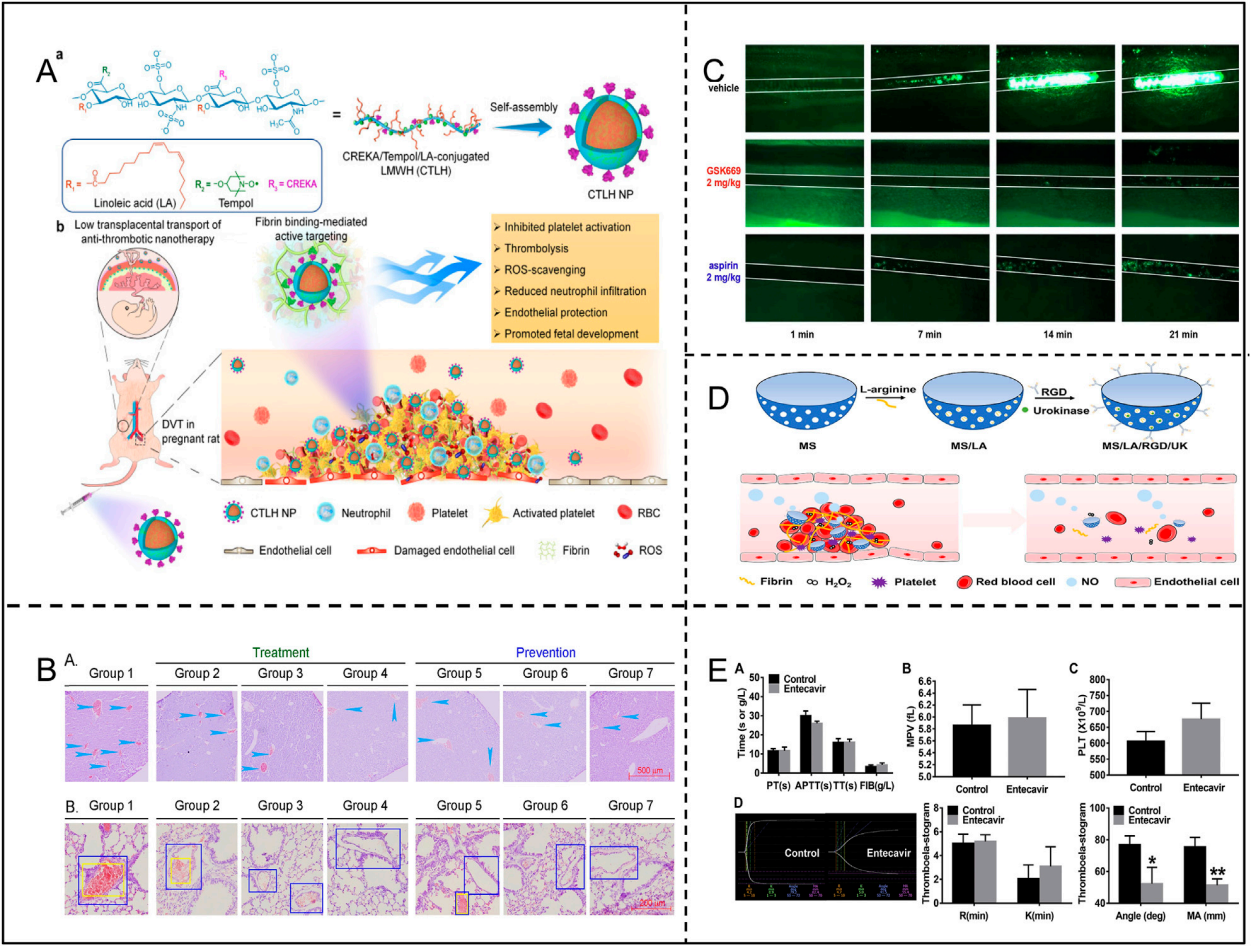
**(A)** Synthesis of CTLH NP and targeting of DVT in pregnant rats. Adapted with permission from Ref. Cheng et al. (2022). Copyright (2022) Springer Nature Limited. **(B)** Long Sheng Zhi Capsules (LSZ) significantly inhibited carrageenan-induced in the vasculature of mouse tissue Thrombosis. Adapted with permission from Ref. Li et al. (2019). Copyright 2019 El Pan et al. (2021) sevier Ltd. **(C)** GSK669 inhibits thrombosis in FeCl-injured mouse mesenteric small arteries. Adapted with permission from Ref. Copyright2020 Elsevier Inc. **(D)** Preparation process of MS/LA/RGD/UK nanomotors and their application in thrombosis therapy. Adapted with permission from Ref. Tao et al. (2022). Copyright 2021 Elsevier Inc. **(E)** Effects of entecavir on the coagulation system, MPV, PLT and TEG. Adapted with permission from Ref. Ming et al. (2020). Copyright 2020 Elsevier B.V.

ROS induce platelet activation and aggregation through multiple mechanisms, significantly promoting thrombosis. Specifically, ROS can directly oxidize key receptors on the platelet surface, such as GPIIb/IIIa integrins, facilitating their activation and tight binding to fibrinogen, which markedly enhances inter-platelet aggregation. Additionally, ROS can activate the phospholipase C (PLC)-calcium ion pathway, resulting in elevated intracellular calcium concentrations. This, in turn, leads to changes in platelet morphology, granule release, and secretion of pro-aggregatory factors, such as ADP and platelet factor 4 (PF4). Moreover, ROS can increase endogenous ROS production through activation of NADPH oxidase and the mitochondrial respiratory chain within the platelet, further amplifying activation signals through a positive feedback mechanism (Walsh et al., 2014). ROS also impair endothelial cell function, reducing the production of anticoagulants such as NO and prostacyclin, thereby indirectly promoting platelet activation. More critically, ROS can increase the release of pro-coagulation factors such as platelet-activating factor (PAF) (Qiao et al., 2018). These mechanisms synergistically promote the pathological process of thrombosis formation under oxidative stress, highlighting the pivotal role of ROS in vascular-related diseases. Notably, researchers Cheng and Zhang et al. constructed a nanoparticle CTLH NP(containing Tempol, a SOD mimetic with ROS scavenging capabilities), which achieved targeted thrombolysis in pregnant rats with deep vein thrombosis (DVT) by inhibiting platelet aggregation, promoting thrombolysis, reducing local inflammation, attenuating oxidative stress, and promoting endothelial repair (Figure 8A; Table 3) (Cheng et al., 2022). Additionally, other studies have found that GSK669, as a NOD2 receptor antagonist, can inhibit ROS production and thrombosis formation by targeting platelet GPVI, thereby reducing thrombus formation in mesenteric arteries of mice. Importantly, GSK669 exerts anti-platelet and antioxidant effects without increasing the risk of bleeding (Figure 8C; Table 3) (Pan et al., 2021).

**TABLE 3:** Therapies against oxidative stress in thrombosis.

Disease	Mechanisms	Treatment	Results	References
Thrombosis	plt activation and aggregation	CTLH NP	Inhibits platelet aggregation, promotes thrombolysis, reduces local inflammation, attenuates oxidative stress, promotes endothelial repair	Cheng et al. (2022)
		GSK669	Targeting platelet GPVI inhibits thrombosis and oxidative stress	Pan et al. (2021)
	Coagulation and Fibrinolytic System	Entecavir	Ameliorates mitochondrial damage in thrombin-activated platelets, prolongs the time to thrombosis, reduces clot weight and length, significantly reduces alpha-angel and MA values in TEG analysis, and prevents thrombosis without affecting the coagulation system, MPV, or PLT.	Ming et al. (2020)
	Inflammatory response	NK	Inhibition of LPS-induced activation of TLR4 and NOX2, and thus the corresponding ROS production, inhibited the activation of MAPKs and the translocation of NF-κB from the cytoplasm to the nucleus	Wu et al. (2020)
	Endothelial cell injury	MS/LA/RGD/UK	LA reacts with ROS to generate NO, which promotes nanomotor movement while stimulating endothelial cell growth, UK thrombolysis	Tao et al. (2022)
		LSZ	Reduces serum P-selectin levels, decreases the expression of TNF-α to MMP2, decreases the expression of platelet receptors (P2Y12, PAR4, and CD36) and the activity of AKT, and reduces the adhesion of human platelets and monocytes to human umbilical vein endothelial cells (HUVECs)	Li et al. (2019)
	RBC aggregation	ABS	Inhibition of pathological aggregation of erythrocytes	ÇİFTÇİLER et al. (2019)

#### 2.3.2 Regulation mechanisms of coagulation and fibrinolytic systems

ROS also profoundly influence the coagulation and fibrinolytic system through multiple mechanisms, promoting thrombosis and aggravating vascular disease. Specifically, ROS can induce the expression of tissue factor (TF) in endothelial cells and monocytes, thereby activating the extrinsic coagulation pathway and increasing thrombin generation (Masuda and Shoko, 2020). Thrombin not only directly promotes fibrin formation, but also enhances platelets and coagulation factors through a positive feedback mechanism, significantly amplifying the coagulation response. Furthermore, ROS can decrease the activity of anticoagulant substances such as antithrombin III (AT-III) and protein C, decreasing their anticoagulant effects and further enhancing coagulation tendency (Walborn et al., 2019). In the fibrinolytic system, ROS inhibit the production of fibrinolytic enzymes by up-regulating fibrinolytic inhibitory factors, such as plasminogen activator inhibitor-1 (PAI-1), preventing fibrin degradation and weakening fibrinolytic function (Robinson et al., 2021). At the same time, ROS also reduce the activity of tissue-type plasminogen activator (tPA), further decreasing plasmin generation, which makes thrombus dissolution more difficult. More seriously, ROS can induce cause endothelial cell damage, reducing the release of anticoagulant and fibrinolytic factors and disrupting the balance between coagulation and fibrinolysis (Syrovets et al., 2012). This mechanism of enhanced coagulation and inhibition of fibrinolysis contributes to a more stable thrombus, significantly increasing the risk of thrombosis (Ekholm and Kahan, 2021; Göbel et al., 2018). Studies have shown that entecavir can inhibit ROS generation by suppressing P2X7R and enhancing SOD activity, which plays an important role in entecavir’s ability to prolong thrombosis and reduce thrombus weight and length (Figure 8E; Table 3) (Ming et al., 2020).

#### 2.3.3 Activation of inflammatory cells

There is a close association between inflammatory response and thrombosis. In an oxidative stress environment, ROS can activate various inflammatory cells, including macrophages, neutrophils, and T cells, triggering the release of inflammatory factors, such as tumor necrosis factor-alpha (TNF-alpha) and interleukins (ILs). These inflammatory factors further promote the activation and aggregation of platelets, enhancing the expression levels of coagulation factors, thereby accelerating thrombus formation. Additionally, inflammatory cells can interact directly with platelets or release extracellular traps (NETs), providing a favorable structure for coagulation and thereby hastening the thrombus development process (J. Chen and Chang, 2022; Wang Y. et al., 2024). Research has revealed that nattokinase (NK) was found to have an inhibitory effect on thrombosis by suppressing lipopolysaccharide (LPS)-induced inflammation and oxidative stress. Specifically, NK reduces ROS production by inhibiting the activation of TLR4 and NOX2 induced by LPS, and inhibits the activation of MAPKs and the translocation of NF-κB from the cytoplasm to the nucleus (Table 3) (Gomez and Owens, 2012; Wu et al., 2023).

#### 2.3.4 Interaction between oxidative stress and vascular endothelium

During thrombus formation, ROS damages endothelial cells through various mechanisms, disrupting their anticoagulant functions and promoting thrombus generation. First, ROS-induced oxidative stress directly damages endothelial cells and disrupts their structural integrity, leading to the exposure of collagen and tissue factor (TF) in the basement membrane after endothelial cell injury. This change activates the coagulation cascade response, which initiates the exogenous coagulation pathway and rapidly increases thrombin generation, which in turn leads to fibrin deposition (Yan et al., 2024; Zheng et al., 2022). Endothelial cell apoptosis also exposes additional procoagulant active surfaces, which further increase platelet adhesion to coagulation factors, thereby exacerbating thrombus formation. Furthermore, ROS inhibit endothelial cell secretion of key anticoagulant substances, including antithrombin III, proteins C and S, and antiplatelet adhesion factors (e.g., NO and prostacyclin PGI2), weakening endothelial cell protection and making platelets more susceptible to adherence to damaged endothelial surfaces. Meanwhile, ROS further enhanced coagulation by activating procoagulant factors such as TF and von Willebrand factor (vWF), increasing platelet adhesion at the site of injury (Hou et al., 2017). In addition, ROS induce endothelial cells to express selectins and adhesion molecules (e.g., ICAM-1, VCAM-1), which enhance the adhesion between platelets and leukocytes and promote local inflammatory responses, thereby exacerbating the process of thrombosis. Li et al. developed a NO-driven silica nanomotor modified with RGD peptide loaded with l-arginine (LA) and urokinase (UK) targets thrombus. (Figure 8D; Table 3) LA reacts with ROS to generate NO, which promotes nanomotor motility, improves drug utilization, and eliminates ROS to alleviate oxidative stress. UK rapidly lyses thrombus, and NO also stimulates the growth of endothelial cells and reduces vascular injury (Tao et al., 2022). Li et al. found that Longshengzhi capsule (LSZ) can significantly inhibit carrageenan-induced thrombosis in mouse tissue vasculature, with its antithrombotic effect associated with the reduction of serum P-selectin level, the reduction of TNF-α and the expression ofMMP2in tissues. Furthermore, LSZ can reduce oxidative stress, lower the expression of platelet receptors (P2Y12, PAR4, and CD36), and decrease AKT activity, thereby reducing the adhesion of human platelets and monocytes to human umbilical vein endothelial cells (HUVECs) (Figure 8B; Table 3) (Kigawa et al., 2014; Li and Kong, 2020).

#### 2.3.5 Involvement of erythrocytes

Erythrocytes are involved in thrombosis by increasing the viscosity of the blood, inducing aggregation of erythrocytes to form “currency columns,” and being physically entrapped into platelets and the fibrin network to increase the density and stability of the thrombus (Bettiol et al., 2022). The erythrocyte antioxidant enzyme system (e.g., SOD, catalase, *etc.*) and non-enzymatic antioxidants (e.g., reduced glutathione, GSH) work together to defend against oxidative stress. However, when these defense mechanisms fail, oxidative stress can have deleterious effects on erythrocytes. Erythrocytes take up ROS from plasma and also produce endogenous ROS themselves *via* NADPH oxidase (Orrico et al., 2023). Endogenous and exogenous ROS induce oxidation of iron in hemoglobin to produce methemoglobin (Fe^3+^), which promotes thrombosis *via* the Fenton reaction that triggers lipid peroxidation, hemolysis, and endothelial cell damage. Oxidative stress damages the erythrocyte membrane and increases the calcium ion permeability of the membrane, leading to the exposure of phosphatidylserine, which in turn activates plasminogen, promotes thrombin generation, and induces thrombosis (Bian et al., 2021). In addition, phosphatidylserine exposure on the erythrocyte membrane releases microbubbles, which have high thrombogenic potential (Yan et al., 2022). Oxidative stress-induced hemolysis of erythrocytes releases free hemoglobin and heme, which can activate the inflammatory response through multiple mechanisms (e.g., NF-κB and TLR signaling pathways) and further enhance thrombosis. In conclusion, oxidative stress promotes the critical role of erythrocytes in thrombosis through a variety of mechanisms (including induction of erythrocyte membrane damage, increased phosphatidylserine exposure, and release of prothrombotic microbubbles). ÇİFTÇİLER et al. found that Ankaferd Hemostat (ABS) inhibits pathological aggregation of erythrocytes and its hemostatic effect is mainly dependent on fibrinogen gamma chain, thrombin and erythrocytes (Table 3) (ÇİFTÇİLER et al., 2019).

Although progress has been made in understanding the effects of oxidative stress on thrombus formation, many aspects remain to be explored in depth. For example, the specific mechanism of action of oxidative stress in different types of thrombosis, as well as its interactions with a variety of components such as platelets, endothelial cells, and erythrocytes, have not yet been fully clarified. Furthermore, accurately assessing and quantifying the levels of ROS in the body, as well as their dynamic changes in different pathological states, remains a significant challenge in current research. Currently, thrombotic therapy relies on anticoagulant and antiplatelet drugs, such as warfarin and aspirin. However, these treatments are associated with bleeding risks, drug resistance, and variable efficacy in different patients. Therefore, it is particularly important to find safe and effective alternative treatment options. Obviously, according to the existing studies, antioxidant measures can effectively inhibit thrombosis, but they also face many problems, such as most of the studies stayed in animal experiments and lacked clinical trial data; the safety, stability, and targeting of nanomaterials need to be further investigated; and the efficacy of traditional drugs is still controversial and lacks the support of large-scale randomized controlled studies. Therefore, exploring and developing more effective antioxidant therapeutic strategies holds significant research value.

### 2.4 Role of oxidative stress in diabetic foot and related therapeutic strategies

**FIGURE 9:**
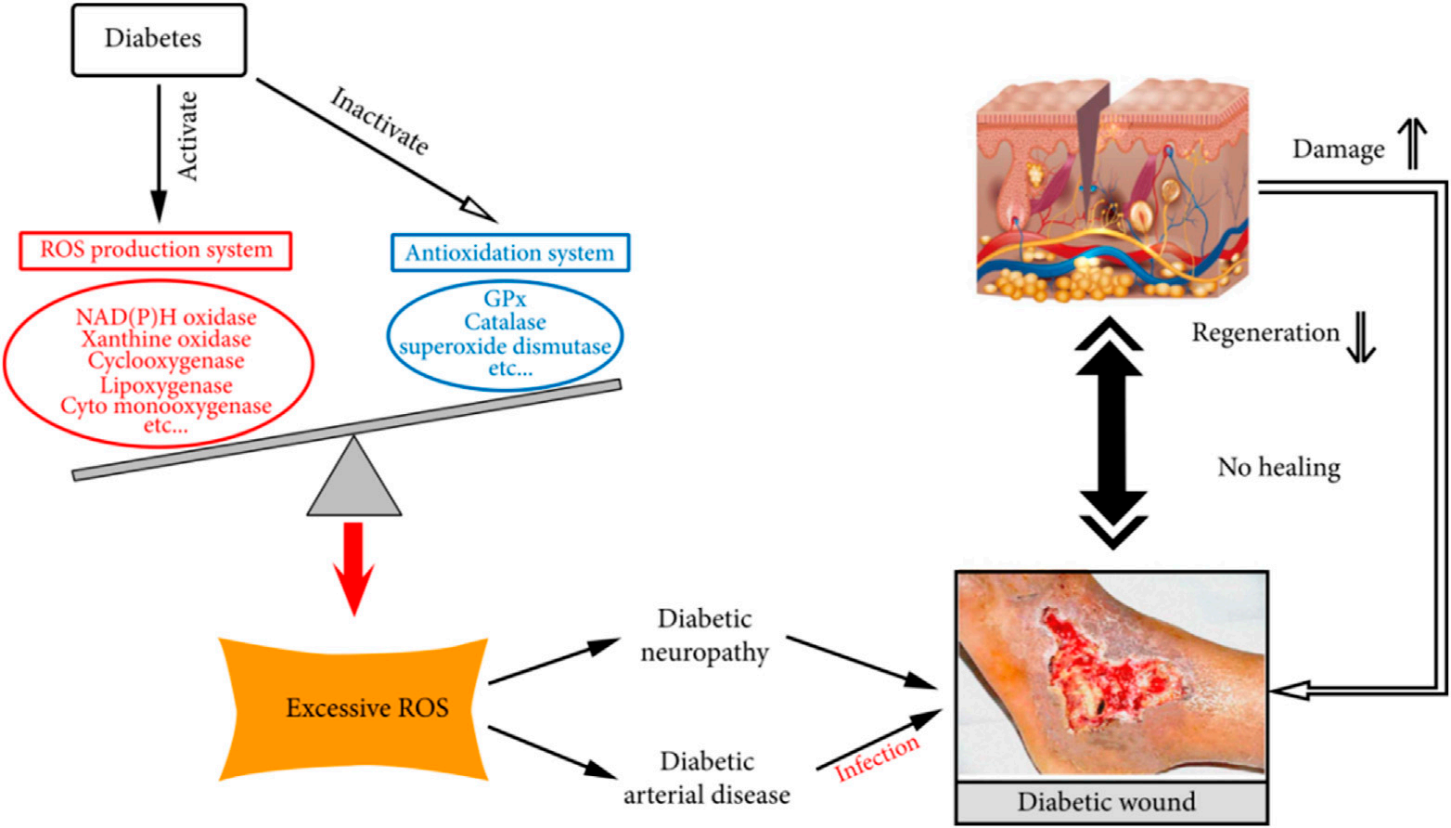
Role of oxidative stress in the development and chronicity of diabetic wounds. Excerpted from reference Deng et al. (2021). Copyright 1999–2024 John Wiley & Sons.

Diabetic foot is a serious complication of diabetes mellitus, which is mainly characterized by foot ulceration, infection and deep tissue destruction, and in severe cases can lead to gangrene and amputation of the foot. The development of diabetic foot is closely related to diabetes-induced chronic hyperglycaemia, peripheral neuropathy and peripheral vasculopathy (Figure 9).

#### 2.4.1 Hyperglycaemia-induced oxidative stress

**FIGURE 10:**
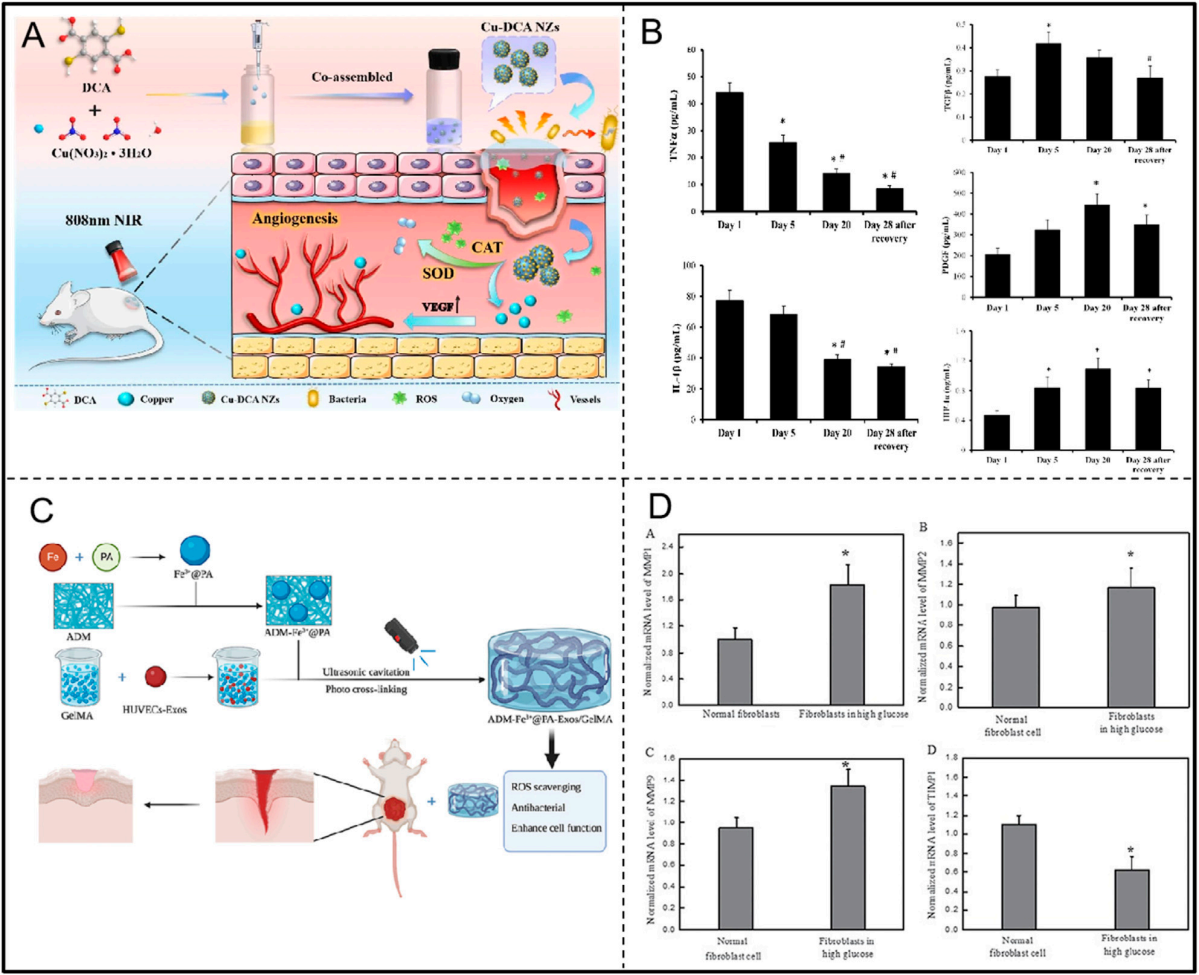
**(A)** Preparation of Cu-DCA NZs with SOD and CAT activities and application in diabetic wound healing. Adapted with permission from Ref. Huang et al. (2023). Copyright (2023) BioMed Central Ltd. **(B)** Changes in inflammatory factors and growth factors during hyperbaric oxygen treatment of the diabetic foot. Adapted with permission from Ref. Capó et al. (2023). Copyright (2023) Int. J. Mol. Sci. **(C)** ADM-Fe3+@PA-Exos/GelMA Hydrogel with Antioxidant and Antimicrobial Properties to Promote Wound Healing in Diabetes. Adapted with permission from Ref. Xiang et al. (2023). Copyright (2023) Elsevier Ltd. **(D)** MDI 301 regulates MMPs and TIMP1 levels to alleviate high glucose-induced skin damage. Adapted with permission from Ref. Zhai and Wang (2017). Copyright (2017) Oncotarget.

Under hyperglycemic conditions, the mitochondrial electron transport chain in the body is active and abnormal, leading to the massive production of superoxide. These superoxides not only triggers endothelial cell dysfunction, but also stimulates increased inflammation (Ceriello, 2005). Simultaneously, the rise in blood concentrations of pro-oxidants such as malondialdehyde (MDA) disrupts the balance between oxidation and antioxidant (PAB) in the body and reduces SOD activity, further worsening the oxidative stress situation (An et al., 2023; Zhao et al., 2019). In addition, the hyperglycaemic environment promotes enhanced activity of aldose reductase and sorbitol dehydrogenase, driving the conversion of intracellular glucose to sorbitol and fructose, a cascade of metabolic abnormalities that impedes the synthesis of myo-inositol in neuronal cells, interferes with the normal conduction of nerve signals, and results in diminished pain and pressure sensation in patients who are less likely to be able to detect foot trauma, increasing the chances of infection (Ceriello, 2005). This series of changes exacerbates the pathologic development of the diabetic foot, making treatment and recovery more challenging. Zhai et al. found that oxidative stress imbalance induced by high glucose concentration plays an important role in the formation of diabetic dermal fibroblasts from normal cells. In addition, this study found that MDI 301 mitigated the effects of high glucose-induced skin damage by balancing oxidative stress and regulating the levels of MMPs and TIMP1 (Figure 10D; Table 4) (Zhai and Wang, 2017).

**TABLE 4:** Therapies against oxidative stress in diabetic foot.

Disease	Mechanisms	Treatment	Results	References
Diabetic foot	Hyperglycaemia	MDI 301	Balancing oxidative stress and regulating MMPs and TIMP1 levels to alleviate high glucose-induced skin damage	Zhai and Wang (2017)
	Vascular damage	Chrysin (LCR)	Promotes angiogenesis, cell migration and anti-apoptosis	Lin et al. (2023)
	Inflammatory response	Hyperbaric oxygen	Increased antioxidant enzymes, decreased pro-oxidant enzymes, inflammatory factors and MMP9, increased growth factors	Capó et al. (2023)
		Cu-DCA NZs	Mimics SOD and CAT activity, reduces wound hypoxia and ameliorates inflammatory accumulation	Huang et al. (2023)
	Reduced tissue repair capacity	ADM-Fe3+@PA-Exos/GelMA	Inhibits bacterial growth and promotes collagen deposition, angiogenesis and maturation in diabetic wounds, while reducing oxidative stress and inflammation	Xiang et al. (2023)

#### 2.4.2 Oxidative stress and the pathological process of diabetic foot

In the pathological process of diabetic foot, there is a close relationship between oxidative stress and endothelial cell damage, forming pathogenic pathway. When the body is in a hyperglycaemic state, excessive ROS are generated through multiple pathways, directly attacking the vascular endothelial cells and damaging their structure and function, thus weakening the natural diastolic capacity of the blood vessels, reducing blood flow, and triggering tissue ischemia and hypoxia (Qu et al., 2022; Zhao et al., 2019). In addition to direct damage to vascular endothelial cells, oxidative stress also hinders the formation of new blood vessels by decreasing the expression of angiogenesis-related factors such as VEGF (vascular endothelial growth factor). Neovascularization is essential for diabetic foot wound healing because new blood vessels provide essential oxygen and nutrients to damaged tissues and help remove metabolic wastes (An et al., 2023; Vujčić et al., 2022).

During the healing process of the diabetic foot, increased oxidative stress negatively affects wound healing by a variety of complex mechanisms. First, during the inflammatory phase, the sustained elevation of blood glucose levels in diabetic conditions leads to the overproduction of intracellular ROS, which are capable of directly damaging cell membranes, proteins, and DNA, thereby impairing cell function and survival. In addition, increased ROS activate multiple intracellular signalling pathways, such as the polyol pathway and protein kinase C (PKC) signalling pathway, which further promote the release of inflammatory factors and activation of inflammatory cells (Accipe et al., 2023b). During the migratory-proliferative phase, oxidative stress has a major impact on endothelial cell function, including a reduction in endothelial-type NO synthase (eNOS) function and a direct decrease in NO bioavailability, leading to endothelial dysfunction. In addition, oxidative stress interferes with keratinocyte and fibroblast function, including affecting the ability of keratinocytes to migrate and proliferate and inhibiting the ability of fibroblasts to proliferate, migrate, and differentiate. These alterations not only slow the wound closure process, but also affect the formation and maturation of new granulation tissue (Accipe et al., 2023a). During the remodelling phase, oxidative stress delays diabetic foot wound healing by affecting collagen deposition and remodelling of the ECM. Increased ROS lead to aberrant synthesis and modification of ECM components, as well as removal of excess cells through apoptosis, and these changes further impede the normal healing process of diabetic foot wounds (H. Zhang et al., 2024). Overall, increased oxidative stress in diabetic foot patients interferes with the normal biological processes of wound healing, from the inflammatory phase to the remodelling phase, where oxidative stress affects cellular function and ECM remodelling through a variety of mechanisms, ultimately leading to a reduced healing capacity of diabetic foot wounds.

Capó et al. found that during the treatment of diabetic foot with hyperbaric oxygen, antioxidant enzymes (CAT and EcSOD) were increased, pro-oxidant enzymes (MPO and XOX), inflammatory markers (TNF-αand IL-1β), and MMP9 were decreased, and growth factors (TGFβ, PDGF, and HIF-1α) were increased, and tended to revert back to their initial values after the wounds were completely healed (Figure 10B; Table 4). And some signalling pathways such as NF-κB, HIF-1 α and MAPKs may play an important role in this (Capó et al., 2023). Xiang et al. developed the ADM-Fe3+@PA-Exos/GelMA hydrogel system to accelerate diabetic wound healing by addressing multiple aspects of the healing process (Figure 10C; Table 4). The hydrogel effectively improves diabetic cell function, inhibits bacterial growth, and promotes collagen deposition, angiogenesis, and maturation in diabetic wounds while reducing oxidative stress and inflammation. Lin et al. first discovered that Lonicerin (LCR) has a strong ability to promote cellular autophagy, and identified sirt1, an upstream regulatory protein, as a potential target of LCR. In addition, LCR was confirmed to promote angiogenesis, cell migration and anti-apoptosis (Table 4) (W. Lin et al., 2022). Huang et al. prepared multifunctional nano-enzymes (Cu-DCA NZs) that effectively mimicked the activities of SOD and CAT. They promoted diabetic wound healing by reducing wound hypoxia and ameliorating inflammation accumulation, accelerating cell proliferation, migration and angiogenesis (Figure 10A; Table 4) (Huang et al., 2023). Therefore, oxidative stress plays a key role in the course of diabetic foot, and the rational application of antioxidants can effectively reduce tissue damage, promote wound healing, and improve disease prognosis.

### 2.5 Role of oxidative stress in thromboangiitis obliterans (Burger's disease)

**FIGURE 11:**
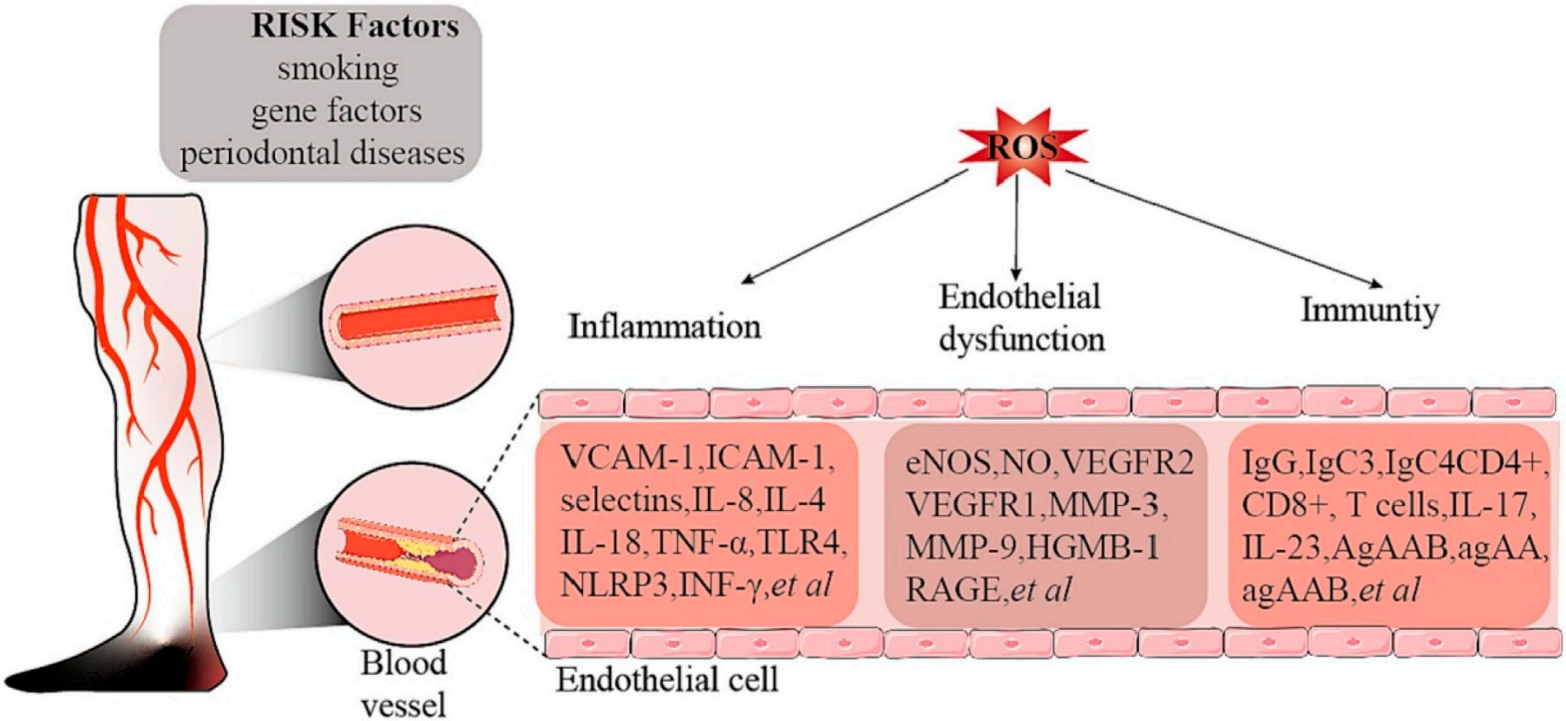
Risk factors and ROS-related mechanisms in thrombotic occlusive vasculitis. Adapted from reference Chen et al. (2023a), 2023 Elsevier Ltd. all rights reserved.

Thromboangiitis obliterans (TAO) is a chronic vascular inflammatory disease primarily affecting small to medium-sized arteries, veins, and nerves, characterized by inflammatory changes in the vessel walls and progressive occlusion. Although the exact etiology of TAO remains unclear, emerging evidence increasingly points to a close relationship with immune pathophysiological mechanisms (Chen et al., 2023b). Among these, oxidative stress occupies a central role in the pathogenesis of TAO, accelerating disease progression and exacerbation through various pathways (Figure 11).

#### 2.5.1 Direct effects of oxidative stress on the vasculature

In comparison to healthy smokers and non-smokers, patients with TAO exhibit significantly elevated levels of oxidative stress (Sun et al., 2017). Oxidative stress activates the nuclear factor-κB (NF-κB) pathway, leading to the induction of NO synthase (iNOS) and an increase in NO production. Excess NO reacts with superoxide anions to form peroxynitrite, which not only triggers lipid peroxidation but also directly impairs endothelial cell function (Sharebiani et al., 2020). Concurrently, there is an upregulation of adhesion molecules and inflammatory factors, further exacerbating vascular inflammation and vascular injury in TAO. In addition, hyperhomocysteinemia, which is more common in patients with TAO, further exacerbates the pathophysiological changes of TAO by activating protease-activated receptors, increasing the production of ROS, and decreasing the bioavailability of NO in the endothelial cells of the cardiac microvasculature, which affects the normal function of the vasculature (Alamdari et al., 2013).

#### 2.5.2 Indirect effects of oxidative stress on the vasculature

The immunopathophysiological basis of TAO has been further demonstrated, particularly by the study of mechanisms such as T-cell-mediated immune injury, IL-33 release, MyD88-dependent TLR signaling pathway, inflammation in the sympathetic ganglia, and inhibition of collateral artery development through IFN-γ and vascular endothelial growth factor receptor 1 (VEGFR1) (Sharebiani et al., 2020). These mechanisms not only reveal the interaction between oxidative stress and immune responses, but also suggest a complex interaction between immune system imbalance and oxidative stress in the pathogenesis of TAO. For example, serum from TAO patients was found to activate human umbilical vein endothelial cells (HUVECs) and alter their adhesion properties, showing a direct link between immune system activation and oxidative stress (Shapouri-Moghaddam et al., 2019). In addition, inflammation of the sympathetic ganglia suggests that the inflammatory response may contribute to the progression of TAO by affecting the nervous system, a process that may also be associated with oxidative stress. The role of oxidative stress in TAO is also revealed in the discussion of the COX inflammatory pathway, particularly through the activation of COX2, which increases the risk of vascular inflammation and thrombosis, which may further exacerbate the effects of oxidative stress. Finally, the mechanism of inhibition of collateral artery development by interferon gamma (IFN-γ) and VEGFR1 suggests that oxidative stress not only affects direct vascular injury, but also interferes with vascular repair and regeneration by affecting key molecules in the process of vascular neovascularization, which provides a new perspective for understanding the complex pathophysiology of TAO (Sun et al., 2017).

Therefore, oxidative stress plays a role in the pathogenesis of TAO through multiple mechanisms, including direct damage to vascular endothelial cells, effects on vascular inflammation and thrombosis, and interference with the process of neovascularization. These findings not only improve our understanding of the pathophysiology of TAO, but also provide possible targets for the development of new therapeutic strategies against TAO.

## 3 Antioxidants in vascular diseases: prevention, clinical evidence, and future directions

Vitamin C, a water-soluble antioxidant, mitigates vascular oxidative stress by neutralizing reactive oxygen species (ROS) in endothelial cells *via* electron donation. It suppresses the Jak2/Stat1/IRF1 signaling pathway, reducing ROS generation and subsequent vascular inflammation (Chaghouri et al., 2021; Du et al., 2022). By enhancing endothelial nitric oxide synthase (eNOS) activity, vitamin C promotes nitric oxide (NO) synthesis, improving endothelial function and inhibiting oxidized low-density lipoprotein (LDL) formation, thereby preventing atherosclerosis initiation (Carr and Maggini, 2017; Ceriello et al., 2013). Additionally, vitamin C reduces vascular smooth muscle cell (VSMC) calcification, with epidemiological studies linking higher dietary intake to a lower risk of abdominal aortic calcification (Jia et al., 2023).

Vitamin E, particularly α- and γ-tocopherol, exerts lipid-soluble antioxidant effects critical for cardiovascular health. α-Tocopherol attenuates oxidative stress by inhibiting NADPH oxidase activity, reducing ROS production, and promoting prostacyclin release (Baez et al., 2017). In contrast, γ-tocopherol enhances endothelial function through eNOS activation, increases NO bioavailability, and scavenges reactive nitrogen species, thereby reducing endothelial injury. It also inhibits platelet aggregation and lowers cholesterol levels, mitigating thrombotic risk (Bielli et al., 2015).

Mineral antioxidants, including selenium, zinc, copper, and manganese, contribute to vascular protection *via* enzymatic pathways. Selenium, a cofactor for glutathione peroxidase (GPx), neutralizes ROS and reduces atherosclerosis risk, as evidenced by a 10-year prospective study (Swart et al., 2018). Zinc acts as a cofactor for superoxide dismutase (SOD), diminishing oxidative stress and inflammation, while copper and manganese support SOD activity to scavenge superoxide radicals (Kitala-Tańska et al., 2024; McCabe and Zhao, 2024).

Despite robust preclinical evidence, clinical outcomes of antioxidants in vascular diseases remain heterogeneous (Table 5). Reduced intestinal flora associated with ALA production has been found in patients with abdominal aortic aneurysms (Ito et al., 2025). However, direct antioxidant effects of ALA in AAA require validation. A 2-year clinical study of carvedilol, a β-blocker with NADPH oxidase inhibitory properties, found no in-stent thrombosis with carvedilol stents, suggesting potential antioxidant and antithrombotic effects (Bae and Jeong, 2012). However, long-term follow-up data on Carvedilol in the treatment of atherosclerosis and thrombosis are limited. Triantafyllos Didangelos’ study found that methylcobalamin improved neurophysiologic parameters, sweating function, and pain scores in patients with diabetic foot (Didangelos et al., 2021). A large prospective cohort study found that diets high in vitamins C and E were associated with a reduced risk of aortic dissection (AAD), but this research study did not confirm a causal relationship (Zeng et al., 2024). HSL calyces exhibit antioxidant properties, enhancing enzymatic and nonenzymatic antioxidant systems in Marfan syndrome patients, reducing oxidative stress, potentially preventing aneurysm formation (Soto et al., 2016). However, direct clinical evidence for HSL treatment of AAA remains lacking. An 8-week randomized controlled study demonstrated that NCB-02 (curcumin-like compounds) lowered levels of inflammatory factors and reduced markers of oxidative stress, improving endothelial function in diabetic patients (Usharani et al., 2008). Simvastatin suppressed NF-κB-mediated ROS in AAA tissues, though clinical evidence remains sparse (Céniga, 2012).

**TABLE 5:** Clinical use of antioxidants.

Antioxidants	Indications	Benefits and effects	References
ALA	AAA	Bacterial genes associated with ALA biosynthesis (M00882, M00883 and M00884, p < 0.0001) were reduced in AAA patients	Ito et al. (2025)
Carvedilol	Thrombosis	No in-stent thrombosis was observed in clinical trials using carvedilol stents during the 2-year clinical follow-up period	Bae and Jeong (2012)
Methylcobalamin	DF	Improvement in all neurophysiologic parameters, sweat function, pain scores	Didangelos et al. (2021)
Vit C\Vit E	AD	Higher dietary Vit C and Vit E intake associated with reduced risk of AAD	Zeng et al. (2024)
HLS	AD	HLS has antioxidant properties that enhance the antioxidant capacity of enzymatic and non-enzymatic systems in the plasma of patients	Soto et al. (2016)
NCB-02 (curcumin-like compounds)	DF	NCB-02 effects on endothelial dysfunction associated with reductions in inflammatory cytokines and markers of oxidative stress	Usharani et al. (2008)
Simvastatin	AAA	Clinical benefits may be related to oxidative stress, blood thrombogenicity, and anti-inflammatory and immunomodulatory properties	Céniga (2012)

Although antioxidants have shown significant vasoprotective effects (e.g., scavenging of reactive oxygen species, reduction of inflammation, improvement of endothelial function, *etc.*) in basic studies, most of the studies are still confined to animal experiments or cellular models, and there is a lack of data from high-quality, rigorously-designed randomized controlled trials (RCTs), which has resulted in insufficient evidence for clinical translation. Meanwhile, some studies have limited sample sizes and lack long-term follow-up data, which may overestimate the clinical benefits of antioxidants and reduce the reliability of study conclusions. Second, current preclinical studies lack standardized criteria for dose setting, dosing method and treatment timing of antioxidants, which makes it difficult to make valid comparisons between different study results. In addition, studies usually do not adequately consider patient heterogeneity, such as age, gender, comorbidities, and underlying medication use, factors that significantly affect the generalizability of study results.

Future research should strengthen the development of high-quality clinical trials, especially RCT design, to further validate the efficacy and safety of antioxidants in the treatment of vascular surgical diseases. Meanwhile, uniform dosage and administration standards should be established, and the precision and generalizability of clinical studies should be improved through subgroup analysis and personalized treatment strategies. In addition, given that antioxidants usually suffer from low bioavailability and short duration of action, novel drug-carrying technologies (e.g., nano-delivery systems) should be actively explored to improve the effectiveness and clinical utility of the drugs.

## 4 Summary and outlook

In summary, we have systematically elucidated the central role of oxidative stress in the pathogenesis of various diseases, including AAD, TAA, thrombosis, diabetic foot, and TAO. We have also explored treatment strategies targeting these pathogenic mechanisms. When the levels of ROS in the intracellular and extracellular environments are abnormally elevated and cannot be promptly cleared, it disrupts the redox balance within cells, triggering a series of biological effects and pathological processes. These include the phenotypic transformation of VSMCs from a contractile to a synthetic state, degradation of the ECM, exacerbation of inflammatory responses, dysfunction of platelets and red blood cells, and imbalance of coagulation and fibrinolytic systems, all of which directly or indirectly promote the occurrence and development of related diseases.

Regarding oxidative stress treatment, edaravone and other antioxidants have demonstrated some efficacy in clearing free radicals and protecting nerve cells; however, their role in peripheral vascular diseases remains to be studied. Common antioxidants like vitamin C, E, and NAC have shown limited efficacy in clinical trials due to low bioavailability and single mechanisms of action. Future research should seek to develop more effective, less toxic novel antioxidants, improve bioavailability, and target the diseased areas more accurately.

Traditional Chinese medicine (TCM) has unique advantages in treating oxidative stress-related diseases. Herbal extracts such as Scutellaria baicalensis (Huangqin) and Salvia miltiorrhiza (Danshen) possess antioxidant, anti-inflammatory, and vascular function-improving properties. Sae-Kwang Ku found that Scutellaria baicalensis significantly protects against high glucose-induced vascular inflammation by reducing vascular permeability, inhibiting the expression of cell adhesion molecules, decreasing pro-inflammatory cytokines, lowering ROS levels, and suppressing the activation of NF-κB (Ku and Bae, 2015). The study by Yinwei Chen et al. also indicates that Scutellaria baicalensis effectively reduces oxidative stress and improves endothelial function and NO generation by decreasing ROS production, inhibiting NADPH oxidase (NOX1), lowering MDA levels, increasing SOD activity, and enhancing NO generation (Y. Chen et al., 2020; Mussbacher et al., 2019). Ji Zhu et al. found that Tanshinone IIA sodium sulfonate (STS) exhibits significant antioxidant and vascular protective effects by reducing ROS generation, lowering lipid peroxidation levels, and inhibiting CLIC1 and its mediated oxidative stress pathways (Zhu et al., 2017). However, TCM also faces challenges such as difficulties in extracting and purifying active components and instability of bioactive compounds.

In recent years, nanomaterials have shown significant potential in treating oxidative stress-related diseases. Novel nanozymes, metal oxide nanoparticles, and polymer-based nanocarriers, with their unique physicochemical properties, allow for targeted drug delivery and controlled release, improving the bioavailability of drugs. For example, the GelMA-CONP designed by Robin Augustine et al. exhibits excellent free radical scavenging activity and promotes diabetic wound healing (Augustine et al., 2020). Meanwhile, the AuNP-MIBI developed by Ludimilla Pereira Tartuce et al. combines the antioxidant properties of gold nanoparticles with the mitochondrial-targeted delivery capability of 2-methoxyisobutylisonitrile (MIBI), providing an efficient and precise drug delivery strategy. This system demonstrates significant antioxidant and cardioprotective effects in the treatment of ischemia-reperfusion (I/R) injury and acute myocardial infarction (AMI) (Tartuce et al., 2020). However, the application of nanomaterials still faces challenges such as *in vivo* metabolism, long-term biocompatibility, and reproducibility and scalability of production. Future research needs to develop highly specific and biocompatible nanomaterials to ensure their *in vivo* stability and safety. Additionally, converting drugs into nanoparticle forms or creating sustained-release nanoparticle capsules may enhance efficacy. Furthermore, increasing research on the toxicity and long-term safety of nanomaterials is crucial for advancing their clinical translation.

However, it is important to acknowledge that ROS play a dual role in physiological processes, and an overly simplified approach to antioxidant therapy may lead to unintended consequences. At low levels, ROS serve as critical second messengers in intracellular signaling, immune defense, and vascular homeostasis. For example, ROS regulate NO synthesis, immune cell activation, and stress-adaptive responses (Forman et al., 2010; Sies, 2017). Excessive removal of ROS may impair normal cellular function, including vascular relaxation, angiogenesis, and immune defense mechanisms. Thus, rather than indiscriminately eliminating ROS, future antioxidant strategies should focus on targeted modulation of oxidative stress while preserving ROS-mediated physiological functions (Marinho et al., 2014).

The dose-dependent and context-specific effects of antioxidant therapy are critical concerns. Large-scale clinical trials, such as Heart Outcomes Prevention Evaluation (HOPE) and Alpha-Tocopherol Beta-Carotene (ATBC), have shown that high doses of certain antioxidants, such as vitamin E and β-carotene, may not provide cardiovascular benefits and, in some cases, may even increase disease risk (Heart Outcomes Prevention Evaluation Study Investigators et al., 2000; Rapola et al., 1998; Willcox et al., 2008). While some antioxidants exhibit protective effects at low doses, they may become pro-oxidative at high concentrations, leading to cellular damage. For example, elevated levels of α-tocopherol can act as a free radical carrier, exacerbating oxidative stress rather than mitigating it (Sotler, 2019). Additionally, conventional antioxidants often lack specificity, indiscriminately scavenging ROS, which may interfere with key cellular signaling pathways and normal physiological functions (Kurutas, 2015). The long-term disruption of redox homeostasis in the body may also lead to metabolic consequences. For instance, prolonged vitamin E supplementation has been associated with an increased risk of insulin resistance, suggesting that excessive antioxidant intervention may impair metabolic stability. The long-term intake of high-dose antioxidants has not consistently demonstrated disease prevention benefits and, in some cases, has been linked to increased health risks. Studies have found that heavy smokers who take large doses of β-carotene over an extended period have a higher risk of developing lung cancer (Kordiak et al., 2022). Moreover, excessive antioxidant consumption may adversely affect mitochondrial function, leading to long-term disruptions in cellular energy metabolism. These findings underscore the necessity of adopting a more precise and personalized approach to antioxidant therapy, taking into account individual oxidative stress levels, metabolic characteristics, and disease progression to optimize therapeutic outcomes while minimizing potential risks.

Despite the promising potential of antioxidant-based therapies, their clinical efficacy remains inconsistent due to challenges in bioavailability, specificity, and safety. Given that ROS play crucial roles in both physiological and pathological processes, it is essential to develop more precise strategies that selectively modulate oxidative stress without disrupting normal cellular functions. Targeted drug delivery *via* nanomaterials, biomarker-guided antioxidant therapy, and combinatorial approaches integrating anti-inflammatory or nitric oxide-regulating agents represent promising directions for future research. Additionally, further exploration into stem cell therapy, gene therapy, and personalized medicine approaches may contribute to a more effective management of oxidative stress-related diseases. Interdisciplinary collaboration and rigorous clinical trials will be essential to translate these strategies into real-world therapeutic applications.

## References

[B1] Accipe L. Abadie A. Neviere R. Bercion S. (2023a). Antioxidant activities of natural compounds from caribbean plants to enhance diabetic wound healing. Antioxidants 12 (5), 1079. 10.3390/antiox12051079 37237945 PMC10215346

[B2] Accipe L. Abadie A. Neviere R. Bercion S. (2023b). Antioxidant activities of natural compounds from caribbean plants to enhance diabetic wound healing. Antioxidants 12 (5), 1079. 10.3390/antiox12051079 37237945 PMC10215346

[B3] Ahmed S. M. U. Luo L. Namani A. Wang X. J. Tang X. (2017). Nrf2 signaling pathway: pivotal roles in inflammation. Biochimica Biophysica Acta (BBA) - Mol. Basis Dis. 1863 (2), 585–597. 10.1016/j.bbadis.2016.11.005 27825853

[B4] Alamdari D. H. Ravari H. Tavallaie S. Fazeli B. (2013). Oxidative and antioxidative pathways might contribute to thromboangiitis obliterans pathophysiology. Vascular 22 (1), 46–50. 10.1177/1708538112473979 23518834

[B5] An Y. Xu B. Wan S. Ma X. Long Y. Xu Y. (2023). The role of oxidative stress in diabetes mellitus-induced vascular endothelial dysfunction. Cardiovasc. Diabetol. 22 (1), 237. 10.1186/s12933-023-01965-7 37660030 PMC10475205

[B6] Augustine R. Zahid A. A. Hasan A. Dalvi Y. B. Jacob J. (2020). Cerium oxide nanoparticle-loaded gelatin methacryloyl hydrogel wound-healing patch with free radical scavenging activity. ACS Biomater. Sci. Eng. 7 (1), 279–290. 10.1021/acsbiomaterials.0c01138 33320529

[B7] Bae I.-H. Jeong M. H. (2012). New drug-eluting stents to prevent stent thrombosis and restenosis for acute myocardial infarction: from the experience of Korean acute myocardial infarction registry. SPIE Proc. 8548, 85483U. 10.1117/12.1000011

[B8] Baez M. del C. Taran M. Scribano M. de L. P. Balceda A. (2017). Inflammatory and oxidative stress markers as indicator of atherogenesis in rats: antioxidants as preventive pharmacological methods. Anti-Inflammatory Anti-Allergy Agents Med. Chem. 16 (2), 87–93. 10.2174/1871523016666170616121133 28618986

[B9] Bettiol A. Galora S. Argento F. R. Fini E. Emmi G. Mattioli I. (2022). Erythrocyte oxidative stress and thrombosis. Expert Rev. Mol. Med. 24, e31. 10.1017/erm.2022.25 36017709 PMC9884766

[B10] Bian Y. Chung H.-Y. Bae O.-N. Lim K.-M. Chung J.-H. Pi J. (2021). Titanium dioxide nanoparticles enhance thrombosis through triggering the phosphatidylserine exposure and procoagulant activation of red blood cells. Part. Fibre Toxicol. 18 (1), 28. 10.1186/s12989-021-00422-1 34348736 PMC8336274

[B11] Bielli A. Scioli M. G. Mazzaglia D. Doldo E. Orlandi A. (2015). Antioxidants and vascular health. Life Sci. 143, 209–216. 10.1016/j.lfs.2015.11.012 26585821

[B12] Bonetti J. Corti A. Lerouge L. Pompella A. Gaucher C. (2021). Phenotypic modulation of macrophages and vascular smooth muscle cells in atherosclerosis—nitro-redox interconnections. Antioxidants 10 (4), 516. 10.3390/antiox10040516 33810295 PMC8066740

[B14] Cao J. Duan S. Zhang H. Chen Y. Guo M. (2019). Zinc deficiency promoted fibrosis via ROS and TIMP/MMPs in the myocardium of mice. Biol. Trace Elem. Res. 196 (1), 145–152. 10.1007/s12011-019-01902-4 31625053

[B15] Capó X. Monserrat-Mesquida M. Quetglas-Llabrés M. Batle J. M. Tur J. A. Pons A. (2023). Hyperbaric oxygen therapy reduces oxidative stress and inflammation, and increases growth factors favouring the healing process of diabetic wounds. Int. J. Mol. Sci. 24 (8), 7040. 10.3390/ijms24087040 37108205 PMC10139175

[B16] Carr A. Maggini S. (2017). Vitamin C and immune function. Nutrients 9 (11), 1211. 10.3390/nu9111211 29099763 PMC5707683

[B17] Céniga M. V. de. (2012). Commentary on ‘simvastatin decreases free radicals formation in the human abdominal aortic aneurysm wall via NF-κB. Eur. J. Vasc. Endovascular Surg. 44 (2), 138. 10.1016/j.ejvs.2012.04.030 22617728

[B18] Ceriello A. (2005). Postprandial hyperglycemia and diabetes complications. Diabetes 54 (1), 1–7. 10.2337/diabetes.54.1.1 15616004

[B19] Ceriello A. Novials A. Ortega E. Canivell S. Sala L. L. Pujadas G. (2013). Vitamin C further improves the protective effect of glucagon-like peptide-1 on acute hypoglycemia-induced oxidative stress, inflammation, and endothelial dysfunction in type 1 diabetes. Diabetes Care 36 (12), 4104–4108. 10.2337/dc13-0750 24130351 PMC3836129

[B20] Chaghouri P. Maalouf N. Peters S. L. Nowak P. J. Peczek K. Zasowska-Nowak A. (2021). Two faces of vitamin C in hemodialysis patients: relation to oxidative stress and inflammation. Nutrients 13 (3), 791. 10.3390/nu13030791 33673687 PMC7997461

[B21] Chen J. Chang R. (2022). Association of TGF-β canonical signaling-related core genes with aortic aneurysms and aortic dissections. Front. Pharmacol. 13, 888563. 10.3389/fphar.2022.888563 35517795 PMC9065418

[B22] Chen Q. Chen J. Li J. Cheng Y. Zhang R. Liu Z. (2023). Recent advances of oxidative stress in thromboangiitis obliterans: biomolecular mechanisms, biomarkers, sources and clinical applications. Thrombosis Res. 230, 64–73. 10.1016/j.thromres.2023.08.015 37639784

[B24] Chen Y. Zhong H. Zhao Y. Luo X. Gao W. (2020). Role of platelet biomarkers in inflammatory response. Biomark. Res. 8 (1), 28. 10.1186/s40364-020-00207-2 32774856 PMC7397646

[B26] Cheng J. Zhang R. Li C. Tao H. Dou Y. Wang Y. (2018). A targeting nanotherapy for abdominal aortic aneurysms. J. Am. Coll. Cardiol. 72 (21), 2591–2605. 10.1016/j.jacc.2018.08.2188 30466517

[B27] Cheng J. Zhang S. Li C. Li K. Jia X. Wei Q. (2022). Functionally integrating nanoparticles alleviate deep vein thrombosis in pregnancy and rescue intrauterine growth restriction. Nat. Commun. 13 (1), 7166. 10.1038/s41467-022-34878-2 36418325 PMC9684510

[B28] Çi̇ftçi̇ler R. Aksu S. Falkmarken N. D. Haznedaroğlu İ. C. (2019). Effects of Ankaferd Hemostat on red blood cell aggregation: a hemorheological study. Turkish J. Med. Sci. 49 (1), 356–360. 10.3906/sag-1808-60 PMC7350838 30761848

[B29] Csiszar A. Wang M. Lakatta E. G. Ungvari Z. (2008). Inflammation and endothelial dysfunction during aging: role of NF-kappaB. J. Appl. Physiol. 105 (4), 1333–1341. 10.1152/japplphysiol.90470.2008 18599677 PMC2576023

[B30] Deng L. Du C. Song P. Chen T. Rui S. Armstrong D. G. (2021). The role of oxidative stress and antioxidants in diabetic wound healing. Oxidative Med. Cell. Longev. 2021, 8852759. 10.1155/2021/8852759 PMC7884160 33628388

[B31] Didangelos T. Karlafti E. Kotzakioulafi E. Margariti E. Giannoulaki P. Batanis G. (2021). Vitamin B12 supplementation in diabetic neuropathy: a 1-year, randomized, double-blind, placebo-controlled trial. Nutrients 13 (2), 395. 10.3390/nu13020395 33513879 PMC7912007

[B32] Du Y.-T. Long Y. Tang W. Liu X.-F. Dai F. Zhou B. (2022). Prooxidative inhibition against NF-κB-mediated inflammation by pharmacological vitamin C. Free Radic. Biol. Med. 180, 85–94. 10.1016/j.freeradbiomed.2022.01.007 35038551

[B33] Eble J. A. Rezende F. F. de. (2014). Redox-relevant aspects of the extracellular matrix and its cellular Contacts*via*Integrins. Antioxid. Redox Signal. 20 (13), 1977–1993. 10.1089/ars.2013.5294 24040997 PMC3993061

[B34] Ekholm M. Kahan T. (2021). The impact of the renin-angiotensin-aldosterone system on inflammation, coagulation, and atherothrombotic complications, and to aggravated COVID-19. Front. Pharmacol. 12, 640185. 10.3389/fphar.2021.640185 34220496 PMC8245685

[B35] Feng H. Wang J.-Y. Yu B. Cong X. Zhang W.-G. Li L. (2019). Peroxisome proliferator-activated receptor-γ coactivator-1α inhibits vascular calcification through sirtuin 3-mediated reduction of mitochondrial oxidative stress. Antioxid. Redox Signal. 31 (1), 75–91. 10.1089/ars.2018.7620 30829051

[B36] Forman H. J. Maiorino M. Ursini F. (2010). Signaling functions of reactive oxygen species. Biochemistry 49 (5), 835–842. 10.1021/bi9020378 20050630 PMC4226395

[B37] Fujii J. Homma T. Osaki T. (2022). Superoxide radicals in the execution of cell death. Antioxidants 11 (3), 501. 10.3390/antiox11030501 35326151 PMC8944419

[B38] Göbel K. Eichler S. Wiendl H. Chavakis T. Kleinschnitz C. Meuth S. G. (2018). The coagulation factors fibrinogen, thrombin, and factor XII in inflammatory disorders—a systematic review. Front. Immunol. 9, 1731. 10.3389/fimmu.2018.01731 30105021 PMC6077258

[B39] Golledge J. (2018). Abdominal aortic aneurysm: update on pathogenesis and medical treatments. Nat. Rev. Cardiol. 16 (4), 225–242. 10.1038/s41569-018-0114-9 30443031

[B40] Gomez D. Owens G. K. (2012). Smooth muscle cell phenotypic switching in atherosclerosis. Cardiovasc. Res. 95 (2), 156–164. 10.1093/cvr/cvs115 22406749 PMC3388816

[B41] Gurung R. Choong A. M. Woo C. C. Foo R. Sorokin V. (2020). Genetic and epigenetic mechanisms underlying vascular smooth muscle cell phenotypic modulation in abdominal aortic aneurysm. Int. J. Mol. Sci. 21 (17), 6334. 10.3390/ijms21176334 32878347 PMC7504666

[B42] Gutmann C. Siow R. Gwozdz A. M. Saha P. Smith A. (2020). Reactive oxygen species in venous thrombosis. Int. J. Mol. Sci. 21 (6), 1918. 10.3390/ijms21061918 32168908 PMC7139897

[B43] Han T. Tang H. Lin C. Yan D. Zhou Z. Yang Y. (2024). Costunolide mitigates inflammation and promotes extracellular matrix integrity of thoracic aortic dissection by inhibiting NF-κB signaling. Int. Immunopharmacol. 131, 111784. 10.1016/j.intimp.2024.111784 38493694

[B44] Hou P. C. Filbin M. R. Wang H. Ngo L. Huang D. T. Aird W. C. (2017). Endothelial permeability and hemostasis in septic shock: results from the ProCESS trial. Chest 152 (1), 22–31. 10.1016/j.chest.2017.01.010 28109962 PMC5577354

[B45] Huang W. Xu P. Fu X. Yang J. Jing W. Cai Y. (2023). Functional molecule-mediated assembled copper nanozymes for diabetic wound healing. J. Nanobiotechnol. 21 (1), 294. 10.1186/s12951-023-02048-1 PMC10464099 37626334

[B46] Hulsmans M. Holvoet P. (2010). The vicious circle between oxidative stress and inflammation in atherosclerosis. J. Cell. Mol. Med. 14 (1–2), 70–78. 10.1111/j.1582-4934.2009.00978.x 19968738 PMC3837590

[B47] Incalza M. A. D’Oria R. Natalicchio A. Perrini S. Laviola L. Giorgino F. (2018). Oxidative stress and reactive oxygen species in endothelial dysfunction associated with cardiovascular and metabolic diseases. Vasc. Pharmacol. 100, 1–19. 10.1016/j.vph.2017.05.005 28579545

[B48] Heart Outcomes Prevention Evaluation Study Investigators Yusuf S. Dagenais G. Pogue J. Bosch J. Sleight P. (2000). Vitamin E supplementation and cardiovascular events in high-risk patients. N. Engl. J. Med. 342 (3), 154–160. 10.1056/nejm200001203420302 10639540

[B49] Ito E. Ohki T. Toya N. Emoto T. Yamashita T. Sugiyama T. (2025). Metagenomic analysis of gut microbiota for abdominal aortic aneurysm. Ann. Vasc. Dis. 18 (1), 24-00105. 10.3400/avd.oa.24-00105 PMC11771153 39877321

[B50] Jeong H. D. Kim J. H. Kwon G. E. Lee S.-T. (2022). Expression of polyamine oxidase in fibroblasts induces MMP-1 and decreases the integrity of extracellular matrix. Int. J. Mol. Sci. 23 (18), 10487. 10.3390/ijms231810487 36142401 PMC9504367

[B51] Jia J. Zhang J. He Q. Wang M. Liu Q. Wang T. (2023). Association between dietary vitamin C and abdominal aortic calcification among the US adults. Nutr. J. 22 (1), 58. 10.1186/s12937-023-00889-y 37964312 PMC10647183

[B52] Jiang Y. Qian H.-Y. (2023). Transcription factors: key regulatory targets of vascular smooth muscle cell in atherosclerosis. Mol. Med. 29 (1), 2. 10.1186/s10020-022-00586-2 36604627 PMC9817296

[B53] Jin S. Kang P. M. (2024). A systematic review on advances in management of oxidative stress-associated cardiovascular diseases. Antioxidants 13 (8), 923. 10.3390/antiox13080923 39199169 PMC11351257

[B54] Kigawa Y. Miyazaki T. Lei X.-F. Nakamachi T. Oguchi T. Kim-Kaneyama J. (2014). NADPH oxidase deficiency exacerbates angiotensin II–induced abdominal aortic aneurysms in mice. Arteriosclerosis, Thrombosis, Vasc. Biol. 34 (11), 2413–2420. 10.1161/atvbaha.114.303086 25189573

[B55] Kitala-Tańska K. Hanć A. Juśkiewicz J. Majewski M. (2024). Prolonged copper supplementation modified minerals in the kidney, liver and blood, and potentiated oxidative stress and vasodilation of isolated aortic rings in young wistar rats. Nutrients 16 (19), 3230. 10.3390/nu16193230 39408198 PMC11478114

[B56] Kordiak J. Bielec F. Jabłoński S. Pastuszak-Lewandoska D. (2022). Role of beta-carotene in lung cancer primary chemoprevention: a systematic review with meta-analysis and meta-regression. Nutrients 14 (7), 1361. 10.3390/nu14071361 35405977 PMC9003277

[B57] Ku S.-K. Bae J.-S. (2015). Baicalin, baicalein and wogonin inhibits high glucose-induced vascular inflammation *in vitro* and *in vivo* . BMB Rep. 48 (9), 519–524. 10.5483/bmbrep.2015.48.9.017 25739393 PMC4641236

[B58] Kuivaniemi H. Ryer E. J. Elmore J. R. Tromp G. (2015). Understanding the pathogenesis of abdominal aortic aneurysms. Expert Rev. Cardiovasc. Ther. 13 (9), 975–987. 10.1586/14779072.2015.1074861 26308600 PMC4829576

[B59] Kurutas E. B. (2015). The importance of antioxidants which play the role in cellular response against oxidative/nitrosative stress: current state. Nutr. J. 15 (1), 71. 10.1186/s12937-016-0186-5 PMC4960740 27456681

[B60] Lai C.-H. Chang C.-W. Lee F.-T. Kuo C.-H. Hsu J.-H. Liu C.-P. (2020). Targeting vascular smooth muscle cell dysfunction with xanthine derivative KMUP-3 inhibits abdominal aortic aneurysm in mice. Atherosclerosis 297, 16–24. 10.1016/j.atherosclerosis.2020.01.029 32059119

[B61] Li Q. Chen Y. Zhao D. Yang S. Zhang S. Wei Z. (2019). LongShengZhi Capsule reduces carrageenan-induced thrombosis by reducing activation of platelets and endothelial cells. Pharmacol. Res. 144, 167–180. 10.1016/j.phrs.2019.04.013 30986544

[B62] Li Z. Kong W. (2020). Cellular signaling in abdominal aortic aneurysm. Cell. Signal. 70, 109575. 10.1016/j.cellsig.2020.109575 32088371

[B63] Lim S. Moon M. K. Shin H. Kim T. H. Cho B. J. Kim M. (2011). Effect of S-adenosylmethionine on neointimal formation after balloon injury in obese diabetic rats. Cardiovasc. Res. 90 (2), 383–393. 10.1093/cvr/cvr009 21245056

[B64] Lin W. Hu K. Li C. Pu W. Yan X. Chen H. (2022). A multi‐bioactive nanomicelle‐based “one stone for multiple birds” strategy for precision therapy of abdominal aortic aneurysms. Adv. Mater. 34 (44), e2204455. 10.1002/adma.202204455 36085560

[B65] Lin Z. Li L. Chen L. Jin C. Li Y. Yang L. (2023). Lonicerin promotes wound healing in diabetic rats by enhancing blood vessel regeneration through Sirt1-mediated autophagy. Acta Pharmacol. Sin. 45 (4), 815–830. 10.1038/s41401-023-01193-5 38066346 PMC10943091

[B66] Liu J. Liu M. Feng J. Zhu H. Wu J. Zhang H. (2022). Alpha-ketoglutarate ameliorates abdominal aortic aneurysm via inhibiting PXDN/HOCL/ERK signaling pathways. J. Transl. Med. 20 (1), 461. 10.1186/s12967-022-03659-2 36209172 PMC9548204

[B67] Lu W.-W. Jia L.-X. Ni X.-Q. Zhao L. Chang J.-R. Zhang J.-S. (2016). Intermedin 1− 53 attenuates abdominal aortic aneurysm by inhibiting oxidative stress. Arteriosclerosis, Thrombosis, Vasc. Biol. 36 (11), 2176–2190. 10.1161/atvbaha.116.307825 27634835

[B68] Lushchak V. I. (2014). Free radicals, reactive oxygen species, oxidative stress and its classification. Chemico-Biol. Interact. 224, 164–175. 10.1016/j.cbi.2014.10.016 25452175

[B69] Marinho H. S. Real C. Cyrne L. Soares H. Antunes F. (2014). Hydrogen peroxide sensing, signaling and regulation of transcription factors. Redox Biol. 2, 535–562. 10.1016/j.redox.2014.02.006 24634836 PMC3953959

[B70] Masuda T. Shoko T. (2020). Clinical investigation of the utility of a pair of coagulation-fibrinolysis markers for definite diagnosis of sepsis-induced disseminated intravascular coagulation: a single-center, diagnostic, prospective, observational study. Thrombosis Res. 192, 116–121. 10.1016/j.thromres.2020.05.009 32473494

[B71] McCabe S. M. Zhao N. (2024). Expression of manganese transporters ZIP8, ZIP14, and ZnT10 in brain barrier tissues. Int. J. Mol. Sci. 25 (19), 10342. 10.3390/ijms251910342 39408669 PMC11476488

[B72] Ming Y. Xin G. Ji B. Ji C. Wei Z. Zhang B. (2020). Entecavir as a P2X7R antagonist ameliorates platelet activation and thrombus formation. J. Pharmacol. Sci. 144 (1), 43–51. 10.1016/j.jphs.2020.07.002 32653340

[B73] Mussbacher M. Salzmann M. Brostjan C. Hoesel B. Schoergenhofer C. Datler H. (2019). Cell type-specific roles of NF-κB linking inflammation and thrombosis. Front. Immunol. 10, 85. 10.3389/fimmu.2019.00085 30778349 PMC6369217

[B74] Nikkel D. J. Wetmore S. D. (2023). Distinctive Formation of a DNA–protein cross-link during the repair of DNA oxidative damage: insights into human disease from MD simulations and QM/MM calculations. J. Am. Chem. Soc. 145 (24), 13114–13125. 10.1021/jacs.3c01773 37285289

[B75] Orrico F. Laurance S. Lopez A. C. Lefevre S. D. Thomson L. Möller M. N. (2023). Oxidative stress in healthy and pathological red blood cells. Biomolecules 13 (8), 1262. 10.3390/biom13081262 37627327 PMC10452114

[B76] Pan G. Chang L. Zhang J. Liu Y. Hu L. Zhang S. (2021). GSK669, a NOD2 receptor antagonist, inhibits thrombosis and oxidative stress via targeting platelet GPVI. Biochem. Pharmacol. 183, 114315. 10.1016/j.bcp.2020.114315 33152345

[B77] Piacenza L. Zeida A. Trujillo M. Radi R. (2022). The superoxide radical switch in the biology of nitric oxide and peroxynitrite. Physiol. Rev. 102 (4), 1881–1906. 10.1152/physrev.00005.2022 35605280

[B78] Piechota-Polanczyk A. Goraca A. Demyanets S. Mittlboeck M. Domenig C. Neumayer C. (2012). Simvastatin decreases free radicals formation in the human abdominal aortic aneurysm wall via NF-κB. Eur. J. Vasc. Endovascular Surg. 44 (2), 133–137. 10.1016/j.ejvs.2012.04.020 22694979

[B79] Qiao J. Arthur J. F. Gardiner E. E. Andrews R. K. Zeng L. Xu K. (2018). Regulation of platelet activation and thrombus formation by reactive oxygen species. Redox Biol. 14, 126–130. 10.1016/j.redox.2017.08.021 28888895 PMC5596263

[B80] Qipshidze N. Tyagi N. Metreveli N. Lominadze D. Tyagi S. C. (2012). Autophagy mechanism of right ventricular remodeling in murine model of pulmonary artery constriction. Am. J. Physiol. Heart Circulat. Physiol. 302 (3), H688–H696. 10.1152/ajpheart.00777.2011 PMC3353777 22101525

[B81] Qiu L. Yi S. Yu T. Hao Y. (2021). Sirt3 protects against thoracic aortic dissection formation by reducing reactive oxygen species, vascular inflammation, and apoptosis of smooth muscle cells. Front. Cardiovasc. Med. 8, 675647. 10.3389/fcvm.2021.675647 34095262 PMC8176563

[B82] Qu K. Yan F. Qin X. Zhang K. He W. Dong M. (2022). Mitochondrial dysfunction in vascular endothelial cells and its role in atherosclerosis. Front. Physiol. 13, 1084604. 10.3389/fphys.2022.1084604 36605901 PMC9807884

[B83] Rapola J. M. Virtamo J. Ripatti S. Haukka J. K. Huttunen J. K. Albanes D. (1998). Effects of alpha tocopherol and beta carotene supplements on symptoms, progression, and prognosis of angina pectoris. Heart 79 (5), 454–458. 10.1136/hrt.79.5.454 9659191 PMC1728686

[B84] Robinson S. Chang J. Parigoris E. Hecker L. Takayama S. (2021). Aqueous two-phase deposition and fibrinolysis of fibroblast-laden fibrin micro-scaffolds. Biofabrication 13 (3), 035013. 10.1088/1758-5090/abdb85 PMC8282251 33440354

[B85] Shapouri-Moghaddam A. Modaghegh M.-H. S. Rahimi H. Ehteshamfar S.-M. Afshari J. T. (2019). Molecular mechanisms regulating immune responses in thromboangiitis obliterans: a comprehensive review. Iran. J. Basic Med. Sci. 22, 215–224. 10.22038/ijbms.2019.31119.7513 31156780 PMC6528722

[B86] Sharebiani H. Fazeli B. Maniscalco R. Ligi D. Mannello F. (2020). The imbalance among oxidative biomarkers and antioxidant defense systems in thromboangiitis obliterans (Winiwarter-Buerger disease). J. Clin. Med. 9 (4), 1036. 10.3390/jcm9041036 32272606 PMC7231233

[B87] Shen X. Xie X. Wu Q. Shi F. Chen Y. Yuan S. (2024). S-adenosylmethionine attenuates angiotensin II-induced aortic dissection formation by inhibiting vascular smooth muscle cell phenotypic switch and autophagy. Biochem. Pharmacol. 219, 115967. 10.1016/j.bcp.2023.115967 38065291

[B88] Sies H. (2017). Hydrogen peroxide as a central redox signaling molecule in physiological oxidative stress: oxidative eustress. Redox Biol. 11, 613–619. 10.1016/j.redox.2016.12.035 28110218 PMC5256672

[B89] Sies H. Berndt C. Jones D. P. (2017). Oxidative stress. Annu. Rev. Biochem. 86 (1), 715–748. 10.1146/annurev-biochem-061516-045037 28441057

[B90] Sies H. (1985). Oxidative stress: introductory remarks. Amsterdam, Netherlands: Elsevier, 1–8. 10.1016/b978-0-12-642760-8.50005-3

[B91] Sotler R. Poljšak B. Dahmane R. Jukić T. Pavan Jukić D. Rotim C. (2019). Prooxidant activities of antioxidants and their impact on health. Acta Clin. Croat. 58, 726–736. 10.20471/acc.2019.58.04.20 32595258 PMC7314298

[B92] Soto M. E. Zuñiga-Muñoz A. Lans V. G. Duran-Hernández E. J. Pérez-Torres I. (2016). Infusion of *Hibiscus sabdariffa L.* modulates oxidative stress in patients with marfan syndrome. Mediat. Inflamm. 2016, 1–12. 10.1155/2016/8625203 PMC4927999 27413258

[B93] Sun X. Law B. Y.-K. Dias I. R. de S. R. Mok S. W. F. He Y. (2017). Pathogenesis of thromboangiitis obliterans: gene polymorphism and immunoregulation of human vascular endothelial cells. Atherosclerosis 265, 258–265. 10.1016/j.atherosclerosis.2017.08.009 28864202

[B94] Swart R. Schutte A. E. Rooyen J. M. van Mels C. M. C. (2018). Selenium and large artery structure and function: a 10-year prospective study. Eur. J. Nutr. 58 (8), 3313–3323. 10.1007/s00394-018-1875-y 30523433

[B95] Syrovets T. Lunov O. Simmet T. (2012). Plasmin as a proinflammatory cell activator. J. Leukoc. Biol. 92 (3), 509–519. 10.1189/jlb.0212056 22561604

[B96] Takaishi K. Kinoshita H. Kawashima S. Kawahito S. (2021). Human vascular smooth muscle function and oxidative stress induced by NADPH oxidase with the clinical implications. Cells 10 (8), 1947. 10.3390/cells10081947 34440716 PMC8393371

[B97] Tanaka A. Hasegawa T. Morimoto K. Bao W. Yu J. Okita Y. (2014). Controlled release of ascorbic acid from gelatin hydrogel attenuates abdominal aortic aneurysm formation in rat experimental abdominal aortic aneurysm model. J. Vasc. Surg. 60 (3), 749–758. 10.1016/j.jvs.2013.07.013 24011462

[B98] Tao Y. Li X. Wu Z. Chen C. Tan K. Wan M. (2022). Nitric oxide-driven nanomotors with bowl-shaped mesoporous silica for targeted thrombolysis. J. Colloid Interf. Sci. 611, 61–70. 10.1016/j.jcis.2021.12.065 34929439

[B99] Tartuce L. P. Brandt F. P. Pedroso G. dos S. Farias H. R. Fernandes B. B. (2020). 2-methoxy-isobutyl-isonitrile-conjugated gold nanoparticles improves redox and inflammatory profile in infarcted rats. Colloids Surf. B Biointerf. 192, 111012. 10.1016/j.colsurfb.2020.111012 32388028

[B100] Usharani P. Mateen A. A. Naidu M. U. R. Raju Y. S. N. Chandra N. (2008). Effect of NCB-02, atorvastatin and placebo on endothelial function, oxidative stress and inflammatory markers in patients with type 2 diabetes mellitus: a randomized, parallel-group, placebo-controlled, 8-week study. Drugs R&D 9 (4), 243–250. 10.2165/00126839-200809040-00004 18588355

[B101] Velarde V. de la Cerda P. M. Jaffa C. D. Arancibia F. Abbott E. González A. (2004). Role of reactive oxygen species in bradykinin-induced proliferation of vascular smooth muscle cells. Biol. Res. 37 (3), 419–430. 10.4067/s0716-97602004000300007 15515967

[B102] Vujčić S. Kotur-Stevuljević J. Vekić J. Perović-Blagojević I. Stefanović T. Ilić-Mijailović S. (2022). Oxidative stress and inflammatory biomarkers in patients with diabetic foot. Medicina 58 (12), 1866. 10.3390/medicina58121866 36557068 PMC9785583

[B103] Walborn A. Rondina M. Mosier M. Fareed J. Hoppensteadt D. (2019). Endothelial dysfunction is associated with mortality and severity of coagulopathy in patients with sepsis and disseminated intravascular coagulation. Clin. Appl. Thrombosis/Hemostasis 25, 1076029619852163. 10.1177/1076029619852163 PMC6714948 31140293

[B104] Walsh T. G. Berndt M. C. Carrim N. Cowman J. Kenny D. Metharom P. (2014). The role of Nox1 and Nox2 in GPVI-dependent platelet activation and thrombus formation. Redox Biol. 2, 178–186. 10.1016/j.redox.2013.12.023 24494191 PMC3909778

[B105] Wang J. Yin Y. Zhang Q. Deng X. Miao Z. Xu S. (2024). HgCl2 exposure mediates pyroptosis of HD11 cells and promotes M1 polarization and the release of inflammatory factors through ROS/Nrf2/NLRP3. Ecotoxicol. Environ. Saf. 269, 115779. 10.1016/j.ecoenv.2023.115779 38056124

[B106] Wang Y. Panicker I. S. Anesi J. Sargisson O. Atchison B. Habenicht A. J. R. (2024). Animal models, pathogenesis, and potential treatment of thoracic aortic aneurysm. Int. J. Mol. Sci. 25 (2), 901. 10.3390/ijms25020901 38255976 PMC10815651

[B107] Willcox B. J. Curb J. D. Rodriguez B. L. (2008). Antioxidants in cardiovascular health and disease: key lessons from epidemiologic studies. Am. J. Cardiol. 101 (10), 75D-86D–S86. 10.1016/j.amjcard.2008.02.012 18474278

[B108] Wu H. Wang Y. Zhang Y. Xu F. Chen J. Duan L. (2020). Breaking the vicious loop between inflammation, oxidative stress and coagulation, a novel anti-thrombus insight of nattokinase by inhibiting LPS-induced inflammation and oxidative stress. Redox Biol. 32, 101500. 10.1016/j.redox.2020.101500 32193146 PMC7078552

[B109] Wu T. Li N. Zhang Q. Liu R. Zhao H. Fan Z. (2023). MKL1 fuels ROS-induced proliferation of vascular smooth muscle cells by modulating FOXM1 transcription. Redox Biol. 59, 102586. 10.1016/j.redox.2022.102586 36587486 PMC9823229

[B110] Xia L. Sun C. Zhu H. Zhai M. Zhang L. Jiang L. (2020). Melatonin protects against thoracic aortic aneurysm and dissection through SIRT1‐dependent regulation of oxidative stress and vascular smooth muscle cell loss. J. Pineal Res. 69 (1), e12661. 10.1111/jpi.12661 32329099

[B111] Xiang K. Chen J. Guo J. Li G. Kang Y. Wang C. (2023). Multifunctional ADM hydrogel containing endothelial cell-exosomes for diabetic wound healing. Mater. Today Bio 23, 100863. 10.1016/j.mtbio.2023.100863 PMC10711188 38089434

[B113] Xu S. Han X. Wang X. Yu Y. Qu C. Liu X. (2024). The role of oxidative stress in aortic dissection: a potential therapeutic target. Front. Cardiovasc. Med. 11, 1410477. 10.3389/fcvm.2024.1410477 39070552 PMC11272543

[B114] Xu T. Wang S. Li X. Li X. Qu K. Tong H. (2021). Lithium chloride represses abdominal aortic aneurysm via regulating GSK3β/SIRT1/NF-κB signaling pathway. Free Radic. Biol. Med. 166, 1–10. 10.1016/j.freeradbiomed.2021.02.007 33588051

[B115] Yan M. Wang Z. An Y. Li Z. Li Y. Zhang H. (2024). OxLDL enhances procoagulant activity of endothelial cells by TMEM16F‐mediated phosphatidylserine exposure. Cell Biol. Int. 48 (6), 848–860. 10.1002/cbin.12150 38444077

[B116] Yan M. Xu M. Li Z. An Y. Wang Z. Li S. (2022). TMEM16F mediated phosphatidylserine exposure and microparticle release on erythrocyte contribute to hypercoagulable state in hyperuricemia. Blood Cells, Mol. Dis. 96, 102666. 10.1016/j.bcmd.2022.102666 35567997

[B117] Yang B. Hu C. Zhang Y. Jiang D. Lin P. Qiu S. (2024). Biomimetic-structured cobalt nanocatalyst suppresses aortic dissection progression by catalytic antioxidation. J. Am. Chem. Soc. 146 (25), 17201–17210. 10.1021/jacs.4c03344 38874405

[B118] Yorn C. Kim H. Jeong K. (2024). Influence of DNA methylation on vascular smooth muscle cell phenotypic switching. Int. J. Mol. Sci. 25 (6), 3136. 10.3390/ijms25063136 38542110 PMC10969885

[B119] Yu Z. Morimoto K. Yu J. Bao W. Okita Y. Okada K. (2016). Endogenous superoxide dismutase activation by oral administration of riboflavin reduces abdominal aortic aneurysm formation in rats. J. Vasc. Surg. 64 (3), 737–745. 10.1016/j.jvs.2015.03.045 26070605

[B120] Zeng C.-R. Gao J.-W. Wu M.-X. You S. Chen Z.-T. Gao Q.-Y. (2024). Dietary vitamin C and vitamin E with the risk of aortic aneurysm and dissection: a prospective population-based cohort study. Nutr. Metabolism Cardiovasc. Dis. 34 (6), 1407–1415. 10.1016/j.numecd.2024.01.024 38664127

[B121] Zhai J. Wang Y. (2017). MDI 301, a synthetic retinoid, depressed levels of matrix metalloproteinases and oxidative stress in diabetic dermal fibroblasts. Oncotarget 8 (27), 43889–43896. 10.18632/oncotarget.16803 28423369 PMC5546448

[B122] Zhang H. Zhang K. Gu Y. Tu Y. Ouyang C. (2024). Roles and mechanisms of miRNAs in abdominal aortic aneurysm: signaling pathways and clinical insights. Curr. Atheroscler. Rep. 26 (7), 273–287. 10.1007/s11883-024-01204-8 38709435

[B124] Zhao K. Zhu H. He X. Du P. Liang T. Sun Y. (2023). Senkyunolide I ameliorates thoracic aortic aneurysm and dissection in mice via inhibiting the oxidative stress and apoptosis of endothelial cells. Biochim. Biophy. Acta (BBA) - Mol. Basis Dis. 1869 (7), 166819. 10.1016/j.bbadis.2023.166819 37499930

[B125] Zhao R. Jiang S. Zhang L. Yu Z. (2019). Mitochondrial electron transport chain, ROS generation and uncoupling (Review). Int. J. Mol. Med. 44, 3–15. 10.3892/ijmm.2019.4188 31115493 PMC6559295

[B126] Zhao Y. Wang L. Liu M. Du A. Qiu M. Shu H. (2023). ROS inhibition increases KDM6A-mediated NOX2 transcription and promotes macrophages oxidative stress and M1 polarization. Cell Stress Chaperones 28 (4), 375–384. 10.1007/s12192-023-01347-8 37140849 PMC10352226

[B127] Zheng D. Liu J. Piao H. Zhu Z. Wei R. Liu K. (2022). ROS-triggered endothelial cell death mechanisms: focus on pyroptosis, parthanatos, and ferroptosis. Front. Immunol. 13, 1039241. 10.3389/fimmu.2022.1039241 36389728 PMC9663996

[B128] Zhu J. Xu Y. Ren G. Hu X. Wang C. Yang Z. (2017). Tanshinone IIA Sodium sulfonate regulates antioxidant system, inflammation, and endothelial dysfunction in atherosclerosis by downregulation of CLIC1. Eur. J. Pharmacol. 815, 427–436. 10.1016/j.ejphar.2017.09.047 28970012

